# Strategy for achieving standardized bone models

**DOI:** 10.1002/bit.27171

**Published:** 2019-10-09

**Authors:** Mikhael Hadida, David Marchat

**Affiliations:** ^1^ Mines Saint‐Etienne, Univ Lyon, Univ Jean Monnet, INSERM, U 1059 Sainbiose Centre CIS Saint‐Etienne France

**Keywords:** bone, in vitro 3D models, perfusion bioreactor, tissue engineering

## Abstract

Reliably producing functional in vitro organ models, such as organ‐on‐chip systems, has the potential to considerably advance biology research, drug development time, and resource efficiency. However, despite the ongoing major progress in the field, three‐dimensional bone tissue models remain elusive. In this review, we specifically investigate the control of perfusion flow effects as the missing link between isolated culture systems and scientifically exploitable bone models and propose a roadmap toward this goal.

## Graphical abstract

The authors highlight the lack of control over the cells’ fluidic environment as a plausible multi‐faceted pitfall in bone tissue engineering. This review offers comprehensive information regarding the influence and mechanics of perfusion flow in a bid to promote the development of more robust in vitro bone tissue models.

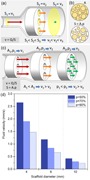

## 1 INTRODUCTION

Animal models remain the current gold standard in preclinical drug screening and proof‐of‐concept studies for innovative treatments, but differences in physiology and metabolism result in low transferability rates for human applications (Scheinpflug et al., 2018). However, major advances in tissue engineering over the past decades have led to the emergence of numerous in vitro organ models dedicated to explorative biology. Regarding bone, the simplest method to obtain bone tissue constructs is to statically culture progenitor cells in porous scaffolds with osteogenic differentiation medium. However, static culture methods rely exclusively on diffusive transfer of soluble substances, such as cell wastes, nutrients, oxygen, and cytokines, creating strong concentration gradients between the fluid‐construct boundary interface and scaffold core (Bancroft et al., 2002; Goldstein, Juarez, Helmke, Gustin, & Mikos, 2001). Depletion of nutrients and waste accumulation in the scaffold core often result in cell death (Keogh, O'Brien, & Daly, 2010; Ratcliffe & Niklason, 2002). Their convective transfer, which is necessary to achieve studies long enough in regard to the duration of the bone modeling cycle in anatomically relevant sized constructs, can be provided by dynamic bioreactors introducing fluid movement (Allori et al., 2008). Extensive information about bioreactors, their various designs (e.g. spinner flasks, rotating wall vessels, mechanical strain, perfusion), and their use can be found in several interesting reviews (Rauh, Milan, Gunther, & Stiehler, 2011; Szpalski, Sagebin, Barbaro, & Warren, 2013; Yeatts & Fisher, 2011). Among those designs, perfusion bioreactors offer the possibility to force the medium through the scaffold, facilitating a more homogeneous environment across the construct volume rather than just improving convection at its surface and provide the best results in terms of overall cell viability and homogeneity (Gaspar, Gomide, & Monteiro, 2012). In addition to controlling and monitoring many culture parameters throughout tissue growth in vitro (e.g., pH, nutrient and waste concentrations), perfusion bioreactors offer a framework to study the role of mechanical cues on cell fate (Bouet, Marchat, Cruel, Malaval, & Vico, 2015). Indeed, although the primary role of perfusion has been to increase the mass transport, the interstitial flow of medium has an additional effect of providing hydrodynamic shear stress, a known regulatory factor of bone development and function (Grayson et al., 2011). Hydrodynamic shear stress (τ in Pa) is the tangential force applied by a fluid on a surface. Any fluid moving along a solid surface will incur shear stress on this surface.

When a mechanical load is applied to bone, interstitial fluid is forced out of the areas of high compressive deformation and flows back when the load is removed (Rauh et al., 2011). In vivo, bone is constantly exposed to stimulation by gravity, muscular contraction, and body movements, generating complex flow patterns that impact cells’ mechanical and chemical environments (Tovar‐Lopez, Dominguez‐Hernandez, Diez‐Garcia, & Araujo‐Monsalvo, 2014). Dynamic bioreactors have shown that these flow effects are paramount to the proper development of bone tissue, positively increasing the expression levels of nitric oxide, prostaglandin E2, and osteoblast‐specific proteins, such as bone sialoprotein, osteopontin, osteocalcin, and type I collagen, along with cell proliferation, distribution, and differentiation (McCoy & O'Brien, 2010; Stiehler et al., 2009; Wittkowske, Reilly, Lacroix, & Perrault, 2016).

Perfusion experiments have been performed since the 1990s (el Haj, Minter, Rawlinson, Suswillo, & Lanyon, 1990; Glowacki, Mizuno, & Greenberger, 1998; Mueller, Mizuno, Gerstenfeld, & Glowacki, 1999) with numerous cell types (e.g., primary cells, cell lines, mesenchymal stem cells; Wittkowske et al., 2016), scaffold materials (e.g., organic, inorganic, metals), scaffold manufacturing techniques (e.g., foaming, salt‐leaching, mesh, bone machining, free‐form shaping; Bouet, Marchat, et al., 2015), and many custom‐made perfusion reactors (Gardel, Serra, Reis, & Gomes, 2014; Gaspar et al., 2012). However, compared to organs such as lung, liver or kidney, in vitro bone tissue models appear to be underdeveloped, with few published models focusing on certain physiological parameters (e.g., load, hypoxia) instead of aiming to comprehensively emulate bone biology (Scheinpflug et al., 2018).

This review aims to offer a broad and multidisciplinary approach to the parameters impacting cell fate in 3D perfused systems to help achieve in vitro bone tissue models with the level of consistency and reliability necessary to the development of functional models suitable for biological and preclinical applications.

## 2 KEY PARAMETERS IN PERFUSION BIOREACTORS

### 2.1 Flow rate, circulation velocity, and shear stress

Perfusion bioreactors supply cells with culture medium at a selected flow rate, which is the volume of medium perfused through the scaffold in a given amount of time. However, cells do not respond directly to flow rate values but to the resulting chemical and mechanical environments.

As stated, the culture medium circulation velocity defines the convective transfer of soluble substances. This velocity defines mass transport rates across the scaffold in association with diffusion phenomena, therefore playing a major role in defining the chemical environment of cells. Regarding mechanical stimuli, they are assumed to be mainly transmitted to bone cells through fluid flow and matrix deformation (Goggin, Zygalakis, Oreffo, & Schneider, 2016; Gusmao & Belangero, 2009; Owan et al., 1997; Paul, Malhotra, & Muller, 2018; You, Weinbaum, Cowin, & Schaffler, 2004). Simplified relationships between flow rate, circulation velocity, and shear stress are reported in the following sections.

#### 2.1.1 Circulation velocity

**Figure 1:**
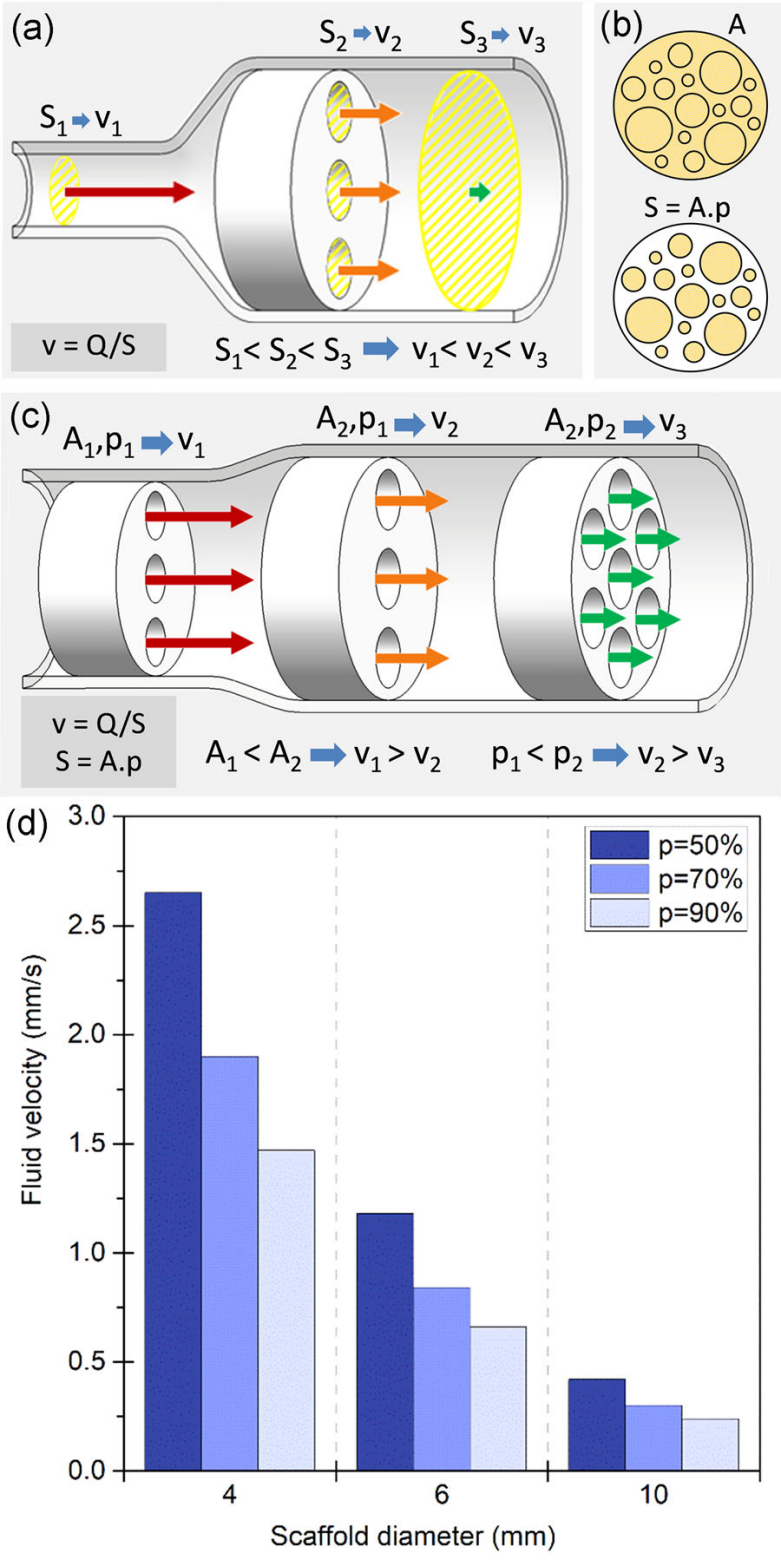
Influence of scaffold properties on flow velocity. (a) For a given flow rate, the fluid velocity (*v*) is inversely proportional to the total open cross‐section (S) it is flowing through, represented in yellow cross‐hatches. (b) The average open cross‐sectional surface (*S)* is determined by the total cross‐sectional area of the scaffold (*A*) and its porosity (*p*). *A* and *S* are successively represented in cross‐hatches. (c) Scaffolds of different sizes and porosities have different open cross‐sectional surfaces, thus generating different velocities for the same flowrate. (d) Average circulation velocity in mm/s for a circular scaffold perfused at a flow rate of 1 ml/min for different scaffold diameters (*D* in mm) and values of porosity (*p*), calculated using Equation (3)

Applied to homogeneous and unidirectional perfusion, the simplified continuity equation states that:

(1) Q=v.S,

where Q is the flow rate and v is the average velocity of the fluid flowing through an open cross‐section of surface S (Figure 1a). The same flow rate will generate different velocities depending on the surface of the open cross‐section it is flowing through.

In a porous scaffold of cross‐section of A and porosity percentage p, the average surface of the open cross‐section along the scaffold (Figure 1b) is defined by the following:

(2) S=A.p.

Thus, the average fluid circulation velocity within the scaffold is directly tied to its dimensions and porosity (Figure 1c) through the relationship:

(3) v=Q/(A.p).

Figure 1d shows the average velocity generated by a 1 ml/min flow rate through circular scaffolds for different common values of scaffold diameter and porosity percentage. For the same flow rate, differences in scaffold size and porosities can easily generate a tenfold difference in average velocity.

#### 2.1.2 Shear stress

**Figure 2:**
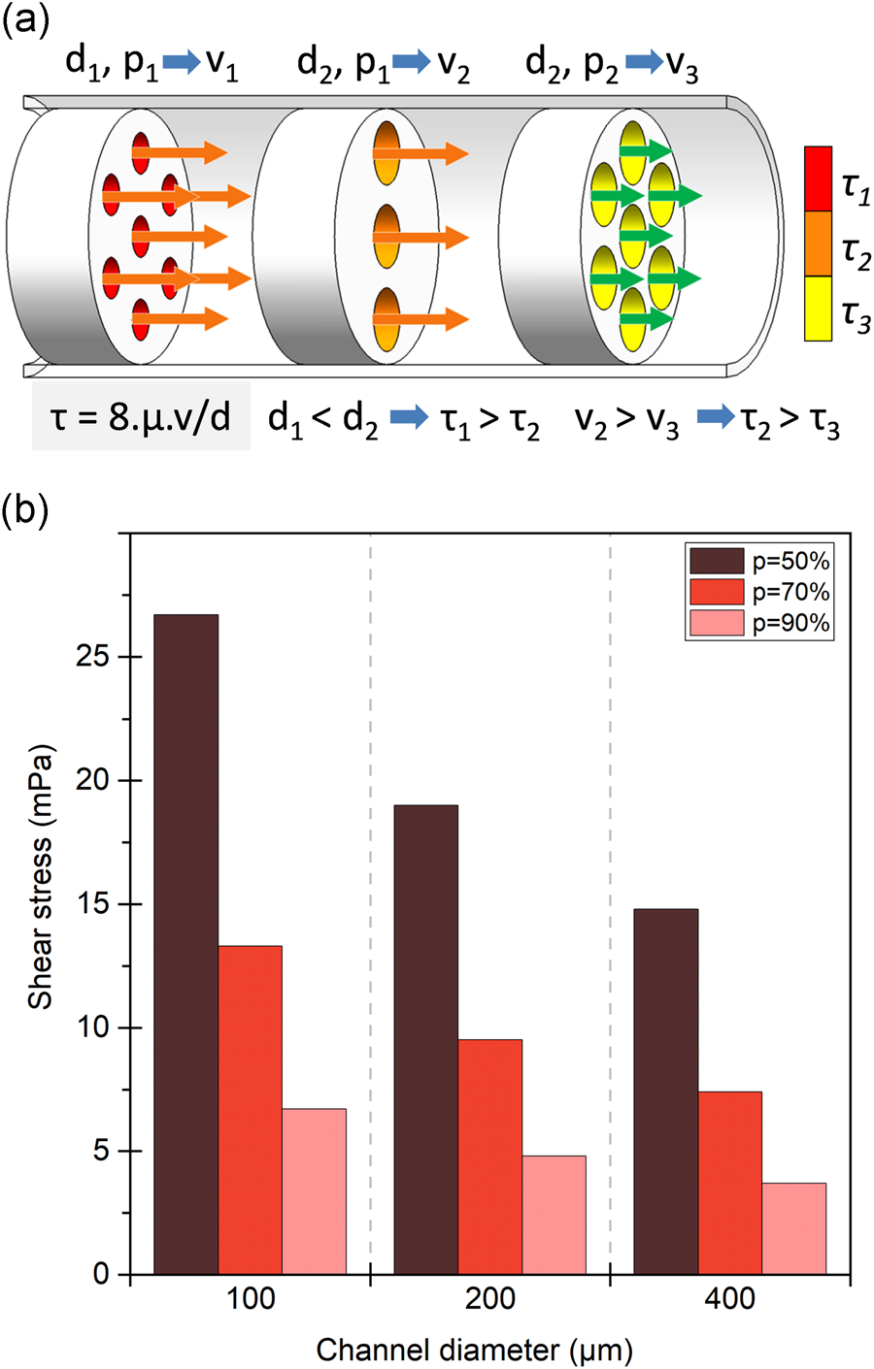
Impact of scaffold properties on flow‐induced shear stress. (a) Shear stress τ inside the channels is proportional to fluid velocity *v* and inversely proportional to the channel diameter *d*. Shear stress is presented in decreasing order from τ_1_ (max) to τ_3_ (min). (b) Approximation of shear stress in mPa in a scaffold of a cross‐section A = 1 cm^2^ perfused at a flowrate of 1 ml/min, and different values of porosity (*p*) and pore diameter (*d* in µm), calculated using Equation (3)

When the thickness of the extracellular matrix (ECM) and cell layers is significantly smaller than the pore diameter, we can use the shear stress applied to the scaffold walls to estimate the flow‐induced shear stress applied to the cells. In cylindrical channels, wall shear stress τ can be extrapolated from the liquid dynamic viscosity μ, the diameter of the channel d (Figure 2a), v through the Poiseuille–Hagen law:

(4) τ=(8.µ.v)/d.

By locally approximating scaffold pores to cylinder fragments, with d being the pore diameter, we obtain the following relationships:

(5) $$\text{τ}\;\text{≅}\;\frac{\text{8.μ.Q}}{\text{d.A.p}},$$

which directly connects local shear stresses to the selected flowrate, medium viscosity, scaffold dimensions, pore sizes, and porosity percentage. Figure 2b shows the average shear stresses generated by a 1 ml/min flowrate through 1 cm^2^ cross‐section scaffolds for different common values of pore diameters and porosity percentage. For this same flowrate, the variations in scaffold internal architecture produce average shear stresses ranging from 3.7 to 26.7 mPa.

### 2.2 Scaffold architectural features defining flow effects

How a given flowrate will translate into mass transport rates and shear stress depends on a combination of the scaffold architectural features and bioreactor characteristics (Du, Ushida, & Furukawa, 2015). The architectural features orienting cell fate by defining flow‐induced mechanical and chemical environments are summarized below.

#### 2.2.1 Shape and dimensions

**Figure 3:**
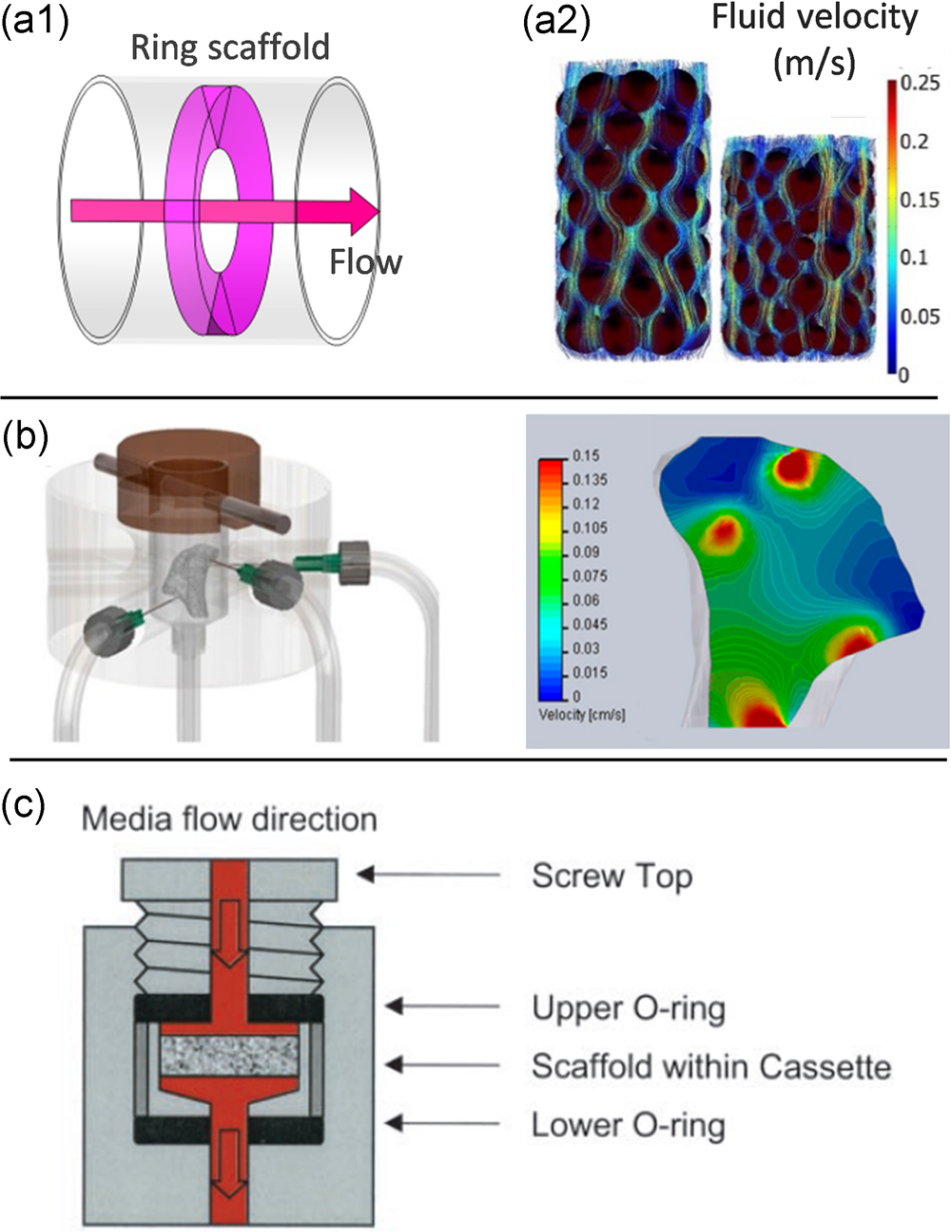
Impact of scaffold and culture chamber designs on flow pathways. Shape and dimensions of the scaffold with respect to the chamber design greatly modify the area and the volume of the scaffold truly perfused; culture medium can flow (a) preferentially all around the scaffold (a1) from the middle (Liu et al., 2012) or (a2) at the periphery (streamline simulation; Cruel et al., 2015), (b) at specific location (Grayson et al., 2010) or (c) through the entire construct (Bancroft, Sikavitsas, & Mikos, 2003)

Scaffolds can range from a few millimeters in size (Grayson et al., 2008; Jagodzinski et al., 2008) to several centimeters (Li, Tang, Lu, & Dai, 2009; Liu et al., 2012), greatly varying the area of the cross‐section exposed to flow. Variations in scaffold shape and bioreactor chamber designs (especially if and how the scaffold is sealed) also define preferential flow pathways. For example, culture medium can sometimes bypass the scaffold porosity (Figure 3a) or be forced in specific flow configurations (Figure 3b). Figure 3c shows a bioreactor where proper perfusion is ensured by press‐fitting the scaffold into a custom silicone cassette.

#### 2.2.2 Porosity

**Figure 4:**
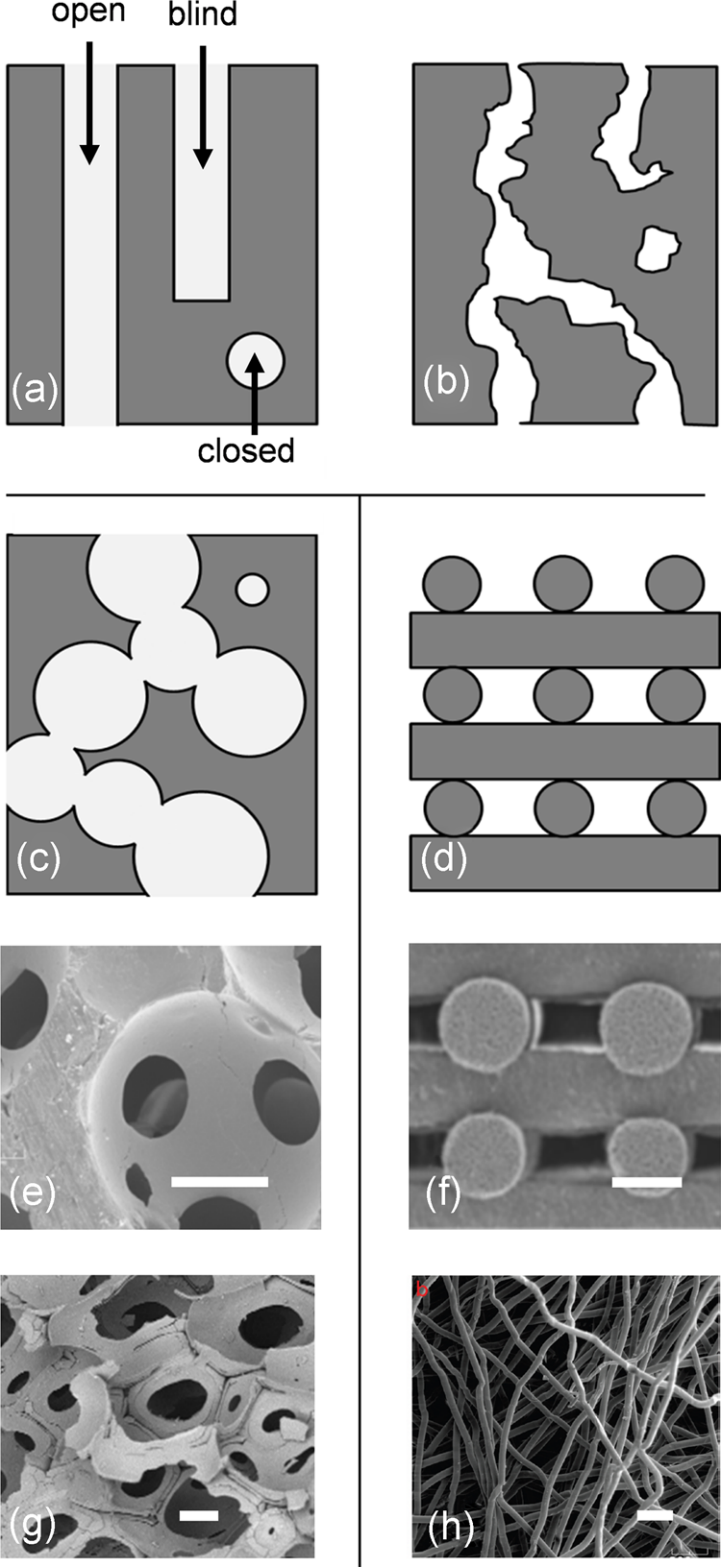
Macroporous structure. Representation of the types of pore space depending on their connection to the surface of the material (open, closed, and blind‐ended; Ashworth et al., 2014) for (a) an ideal structure composed of channels, (b) a tortuous porous network and a scaffold composed of (c) spherical interconnected pores or (d) rods. (e) and (g) show corresponding images of scaffolds composed of interconnected macropores (obtained by PMMA skeleton and organic foam impregnation, respectively), (f) and (h) show scaffolds composed of intertwined fibers obtained by robocasting (from Martinez‐Vazquez, Pajares, Guiberteau, and Miranda, 2014) and fiber spunbonding (from VanGordon et al., 2011). respectively. Scale bar = 200 µm

The macroscopic structure produced by a network of pores is often described using porosity values, expressed as a percentage of the volume of voids over the total volume of the scaffold and often ranging from 50% to 90% (Gariboldi & Best, 2015). Porosity can be open, closed, or blind‐ended (Figure 4a). However, only open porosity is directly conducive to tissue in growth. Porosity is only one of the numerous parameters that may be used to describe the porous architecture of a scaffold (e.g., interconnectivity, pore orientation, tortuosity, pore and interconnection shape). Used alone, porosity is a poor predictor of biological responses. In particular, mass transport and shear stress values, which are key factors affecting cell fate and tissue development, cannot be evaluated based on the porosity value alone; other architectural parameters must be provided (Ashworth, Best, & Cameron, 2014; Bohner, Loosli, Baroud, & Lacroix, 2011).

#### 2.2.3 Macropore size and geometry

As stated in Section 2.1, for a given velocity, shear stress is inversely proportional to the channel diameter (*d*). Therefore, pore size is a significant parameter to know and control (if possible) to determine the relationship between cell behavior and flow effects. Unfortunately, mainly due to the manufacturing techniques used to produce scaffolds, their porous architectures are always more complex than an arrangement of straight channels, as schematized in Figure 4b–d (Bouet, Marchat, et al., 2015). Random macropore distribution, size, orientation, shape, and so forth are predominant in scaffolds used for bone tissue engineering (BTE), which mainly consist of interconnected pores (Figure 4e,g) or intertwined fibers (Figure 4f,h). Macroporosity is almost systematically approximated as spherical with a unique dimension, the “mean diameter,” or in the best case, a diameter distribution (Bohner et al., 2011). This simplification is not representative of the actual macropore geometry and does not include the interconnection features, which are defining parameters for local circulation velocities and shear stresses.

## 3 FLOW EFFECTS

### 3.1 Significance of flowrate

Perfusion studies introduced flowrate as a defining parameter of cell behavior. Studies exploring different flowrates in a setup show, first that cell responses to flow perfusion is value dependent (Bancroft et al., 2002; Cartmell, Porter, Garcia, & Guldberg, 2003; Grayson et al., 2008; Li et al., 2009; Sonnaert et al., 2017), and second that cells can be surprisingly sensitive to moderate variations in flowrate (Cartmell et al., 2003; Su, Wang, & Chou, 2014). Bancroft et al. (2002) cultivated rat marrow stromal osteoblasts in perfused fiber mesh titanium scaffolds and found that an increase in flowrate from 0.3 ml/min to 1 ml/min generated an over six‐fold increase in the calcium content of cultured scaffolds. Conversely, in Cartmell et al. (2003), increasing the flowrate from 0.1 to 0.2 ml/min caused a four‐fold decrease in the total DNA. These studies illustrate that cell behavior, especially viability and differentiation rates, can be significantly altered by subtle changes in flowrates.

**Figure 5:**
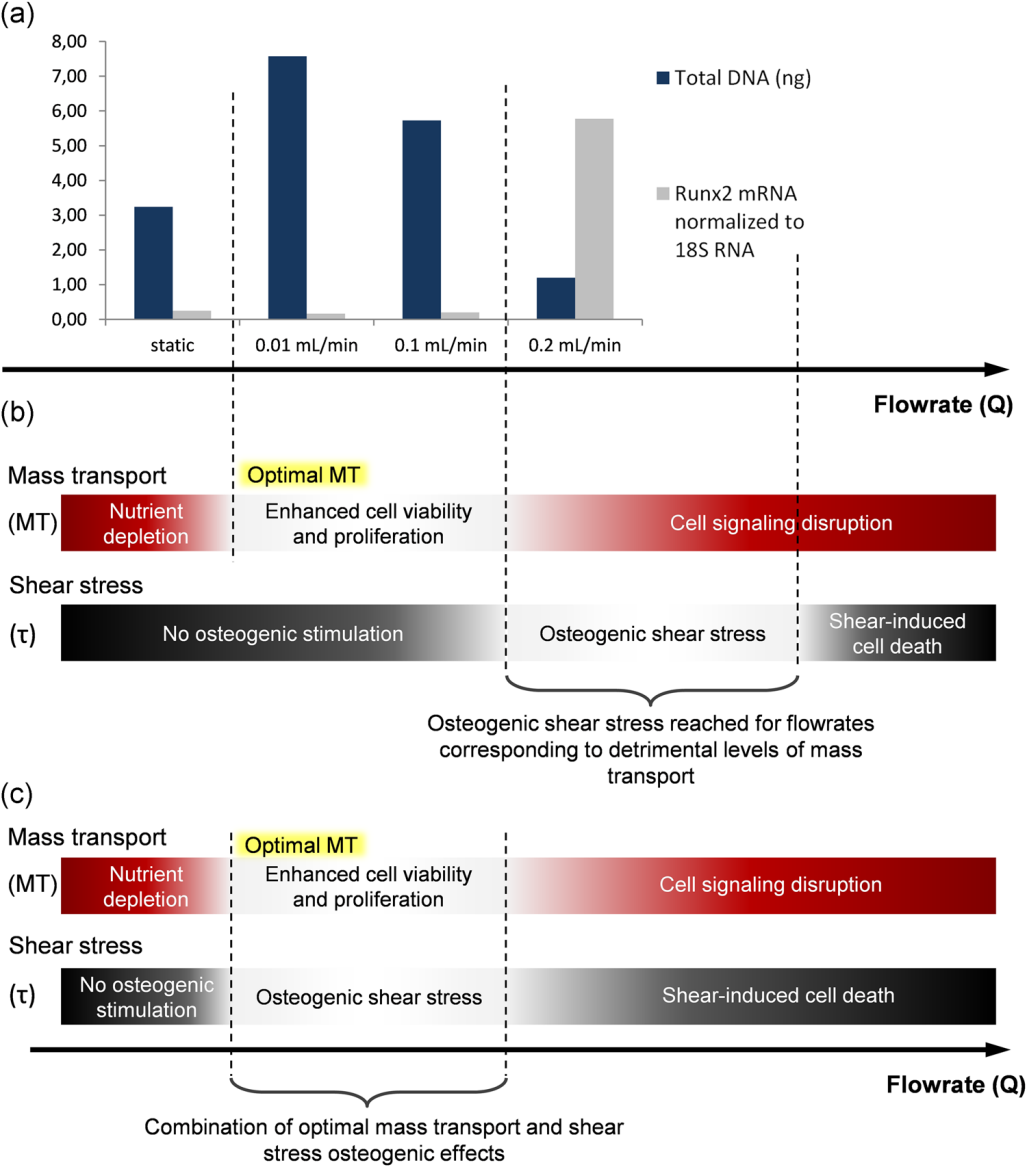
Influence of the combination of shear stress and mass transport on cells fate. (a) Total DNA content (cell proliferation) and Runx2 gene expression measured after 7 days of culture of MC3T3‐E1 immature osteoblast‐like cells in human trabecular bone scaffold under static or dynamic medium flow conditions (from Cartmell et al., 2003). (b) The existence of thresholds for mass transport rates and shear stresses visualized here could explain the marked changes observed between perfusion flowrates of 0.1 and 0.2 ml/min. (c) Appropriate levels of shear stress can have significant osteogenic effects, but higher values cause cell damage and detachment (McCoy & O'Brien, 2010). In this hypothesis, achieving higher shear stresses at a given flowrate would allow benefiting simultaneously from the osteogenic effects of shear stress and mass transport

Cell behavior is not related to flowrate in a linear manner. In the same study by Bancroft et al. (2002), increasing the flowrate from 1 ml/min to 3 ml/min “only” doubled the calcium content. In Cartmell et al. (2003), no significant change in DNA content or OCN and Runx2 expression was observed between 0.01 ml and 0.1 ml/min, whereas a sharp decrease in DNA and an increase in OCN and Runx2 were observed at 0.2 ml/min (Figure 5a). These results suggest that cells are particularly responsive to ranges and thresholds of stimuli.

### 3.2 Confusion surrounding flowrate

Given the significance of flowrate values, a priority in the field of in vitro BTE is determining the “optimal flowrate” for osteogenesis. However, as described in Section 3.1, studies aiming to assess flowrate effects obtain contradictory results regarding the optimal perfusion rate and describe different effects for identical flowrates. For instance, using a static culture as a control, Gomes, Holtorf, Reis, and Mikos (2006) and Jaasma & O'Brien (2008) describe a significant increase and significant decrease in the cell population for a 1 ml/min flowrate, respectively.

On the whole, the flowrate value remains a confounding variable scattered across four orders of magnitude (from 2 µl/min; Bouet, Cruel, et al., 2015 to 60 ml/min; Chen et al., 2016) without clear patterns between the selected flowrate and observed osteogenic effects. Selected flowrates in perfusion studies do not necessarily rely on experimental design and are often seemingly arbitrarily fixed to 0.1 or 1 ml/min (Allori et al., 2016; Gardel et al., 2014; Gomes et al., 2006; Holtorf, Datta, Jansen, & Mikos, 2005; Jaasma & O'Brien, 2008; Sinlapabodin, Amornsudthiwat, Damrongsakkul, & Kanokpanont, 2016; Van Gordon et al., 2011) or refer to different setups. For example, Baas, Kuiper, Yang, Wood, and El Haj (2010) quote one of their articles from 1990 to support the choice of a 0.1 ml/min flowrate, suggesting that this flowrate may have provided consistently good results over two decades. However, the scaffolds used in Baas et al. are of different dimensions and internal architecture than those in their reference study (Baas et al., 2010; el Haj et al., 1990).

As explained in Section 2.1, for a given flowrate, differences in the scaffolds architecture result in different local fluid speeds and shear stresses. The combined influence of both should be systematically considered when interpreting perfusion study results. In Cartmell et al. (2003), the upregulated expression of OCN and Runx2, which are characteristic responses to shear stress exposure (Wittkowske et al., 2016), was observed at 0.2 ml/min (Figure 5a). According to the authors, the sharp decrease in cell viability also observed for this flowrate may be linked to the increased shear stress. However, the osteogenic levels of shear stress are not usually correlated with a decrease in cell viability (Bancroft et al., 2002; Chen et al., 2016; Farack et al., 2011; Grayson et al., 2008; Holtorf, Sheffield, Ambrose, Jansen, & Mikos, 2005; Kleinhans et al., 2015; Li et al., 2009; Liu et al., 2012; Su et al., 2014), and we hypothesize that the shear stress level required to elicit an osteogenic cell response in the scaffolds used is reached only for flowrates inducing mass transport levels already detrimental to cells (Figure 5b). Although relevant mass transport rates enhance cell viability and proliferation, excessively high rates have inhibitory effects that may be linked to cell signaling disruptions (Grayson et al., 2011; Li et al., 2009). This idea would be consistent with the structure of the scaffolds used, which have a relatively high porosity (82%) and an unusually large mean pore size (645 µm), resulting in lower shear stresses at a given flow rate (see Section 2.1). Based on this observation, the protocol parameters could be adjusted by using scaffolds with a smaller pore size or a more viscous culture medium (*c.f*. Section 5.1.3) to obtain a better combination of shear stress and mass transport effects, as proposed in Figure 5c.

Therefore, approaching flowrate in terms of resulting circulation velocities and shear stresses would offer additional insight into result interpretation and understanding why perfusion can reduce cell viability (Bartnikowski, Klein, Melchels, & Woodruff, 2014; Cartmell et al., 2003; Jaasma & O'Brien, 2008; McCoy, Jungreuthmayer, & O'Brien, 2012). Similarly, studies investigating optimal scaffold pore sizes and porosity (Chen et al., 2016; Gomes et al., 2006; McCoy et al., 2012) in perfusion bioreactors should consider that the observed results may not actually be only related to the scaffold features but also to the corresponding shear stress and mass transport resulting from the perfusion of this scaffold at the selected flow rate (see also Section 4.4.1). Thus, a challenging target in BTE is the determination of these optimal shear stress and circulation velocity ranges for a given cell type.

## 4 OBSTACLES IN DEFINING OPTIMAL FLOW EFFECTS

In the context of the ongoing pursuit of optimal operating conditions that will yield the desired levels of tissue performance or functioning, the determination of optimal shear stress and circulation velocity ranges for a given cell type constitutes both a challenging target and a major potential milestone in BTE.

### 4.1 Using reference shear stress values

Fixing or identifying optimal shear stress and circulation velocity in complex 3D systems from approximated variables only (e.g., pore size, flow homogeneity in the scaffold) is arduous. Therefore, the BTE community primarily relies on different values from the literature.

#### 4.1.1 Biomimetics

**Figure 6:**
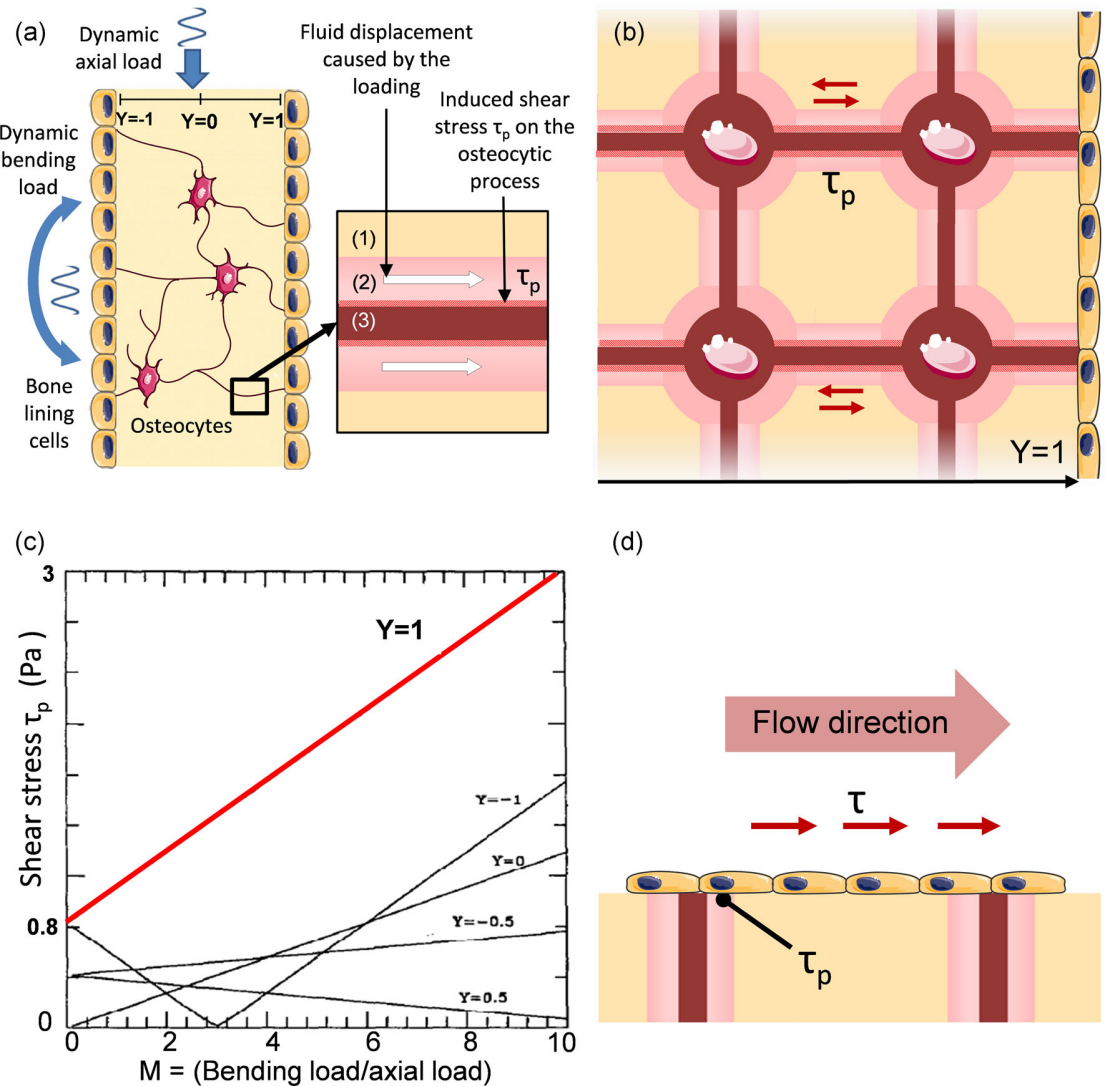
Weinbaum lacunar–canalicular porosity model. (a) Diagram showing a simplified trabecular cross‐section with bone lining cells and osteocytes submitted to oscillatory axial and bending loads as studied by Weinbaum et al. (1994). The fluid displacement caused by the loading in the periosteocytic space (1) of the canaliculi (2) generates shear stress on osteocytic processes (3). (b) Scheme illustrating the idealized Weinbaum's model of a trabecular cross‐section where the shear stress amplitude on the membrane surface of the osteocytic processes τ_p_ is calculated for different depths Y. (c) Plot of the maximum τ_p_ as a function of the bending to axial load ratio at different locations Y. The known 0.8–3 Pa range (in red) corresponds to the periodic maxima at Y = 1 for M ranging between 1 and 10 (from Weinbaum et al., 1994). (d) Difference between τ_p_ at Y = 1 and the shear stress τ applied to the cell surfaces

In 1994, Weinbaum, Cowin, and Zeng (1994) proposed a model for the in vivo mechanical excitation of osteocytes in which physiological shear stresses have been determined in the range of 0.8–3 Pa. Despite more recent models (Min, Lee, Lee, & Hong, 2018; Wu et al., 2016), this now well‐known range has become the reference value for in vivo shear stress BTE. In this model, a trabecula is submitted to a combination of axial and bending dynamic loads, causing displacement of intracanalicular fluid, which in turn induces shear stress τ_p_ along the membrane of osteocytic processes (Figure 6a). To estimate the range of shear stress at osteocyte membranes (dendrite and cell body), the authors proposed a simplified model of the mineralized matrix, representing only the lacunar‐canalicular porosity as a periodic unit cell (Figure 6b). In this model, the 0.8–3 Pa range corresponds to the loading cycle maximum shear stress on the osteocyte membrane close to the osteon wall (Y = 1), that is, where the deformation is maximal, for different load cycle combinations (see Figure 6c).

The shear stresses calculated in most perfusion studies correspond to flow‐induced shear stress on scaffold walls, which would correspond to the shear stress applied to osteon walls in vivo. Reproducing the theoretical 0.8–3 Pa range in perfusion studies is equivalent to trying to reproduce the physiological shear stress τ_p_ applied to osteocyte membranes approaching the osteon walls by applying a similar shear stress on scaffold walls (Figure 6d). This configuration neither emulates the in vivo stimulation of bone lining cells (i.e., flat osteoblastic cells covering bone surfaces) nor the mechanical environment encountered by osteocytes in the lacunar‐canalicular porosity (Wittkowske et al., 2016). Therefore, there is a priori no scientific incentive to reproduce this range of shear stress in perfusion experiments.

#### 4.1.2 Two‐dimensional systems

**Figure 7:**
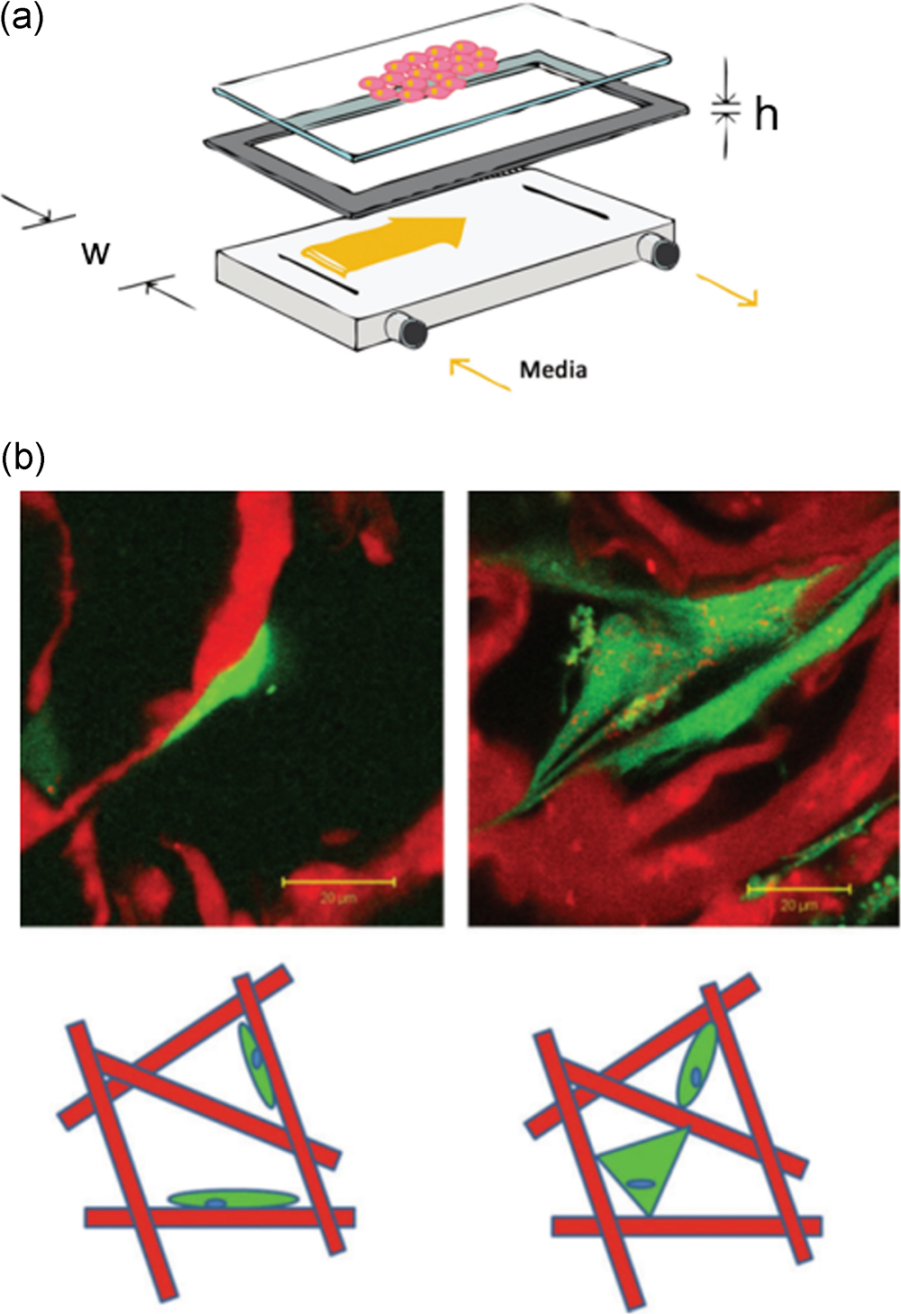
Two‐dimensional models and consequence on cell attachment. (a) Schematic of a parallel‐plate flow chamber (from Cooper et al., 2012) (b) Top: fluorescence microscopy images showing cells attached either predominantly flatly to collagen struts (left) or in a bridged manner (right). Bottom: schematic diagram of attachment morphologies with flatly attached cells on the left and bridged (either dual or multiattachment points) cells on the right. For both microscopy images and schematics, the collagen structure is depicted in red, and the cell cytoplasm is in green (from McCoy and O'Brien, 2010)

Parallel flow chambers were specifically introduced to study shear stress effects. In a rectangular section, wall shear stresses are reliably defined by the following equation:

(6) τ=(6×Q×μ)/(w×h²)

with *Q* being the flowrate, *µ* being the medium viscosity, *w* being the width, and *h* being the height of the flow chamber (Figure 7a).

In these devices, the shear stresses exerted on cells are approximately equal to the chamber wall shear stresses, allowing fine tuning of these parameters. Two‐dimensional (2D) plates are a powerful tool in mechanotransduction studies as they allow accurate experimental designs built around controlled shear stress values. However, 2D culture conditions do not reproduce the typical environment of bone cells as they are forced to grow in monolayers (Antoni, Burckel, Josset, & Noel, 2015). An artificial flat and rigid surface is a geometrical, mechanical environment that affects the cytoskeleton (e.g., actin patterns) of bone cells and more broadly, their fate (Dalby, Gadegaard, & Oreffo, 2014; Zhou et al., 2017). Consequently, in contrast to 3D setups, 2D configurations notoriously skew bone cell responses to mechanical stimuli (Juignet et al., 2017; McCoy & O'Brien, 2010).

In macroporous scaffolds (3D environment), cells can either be attached flatly to the scaffold surface or bridged between two or more surfaces (Figure 7b; Annaz, Hing, Kayser, Buckland, & Di Silvio, 2004). Bridged cells are expected to experience upto 500 times greater levels of cytoskeletal deformation at an equal shear (Jungreuthmayer et al., 2009). For this reason, osteogenic shear stress obtained in 2D conditions cannot be used as a reference to predict cell behavior in 3D porous scaffolds.

#### 4.1.3 Deducing optimal ranges from the literature

Numerous fluid shear stress values have been reported as osteogenic. Values as low as 10^−4^ Pa and upto 2 Pa have yielded positive results in 3D systems (Chen et al., 2016; McCoy & O'Brien, 2010). In the absence of means to compare the osteogenicity of shear stress values in different setups, it is possible that “popular” shear stress ranges merely correspond to protocols commonly found in the literature. Moreover, as most reported studies used static culturing as a control, the effects attributed to shear stress values can result from increased circulation rates. As both shear stress and circulation velocity are tied to flowrate, perfusion studies based on flowrate variation do not permit us to distinguish which cell responses are associated with velocity‐regulated chemical cues and which are correlated with the biomechanical cues imparted by proportionally modulated shear stresses.

This problem persists in studies that adequately report both shear stress and circulation velocity ranges. For instance, Grayson et al. (2011) noted the impracticality of using flowrate values as a parameter and instead reported flow velocities, shear stresses, and oxygen concentrations inside decellularized bone scaffolds, providing useful data points. However, controlling velocity by changing the flowrate proportionally altered shear stress values. The observed effects are then tied to the resulting velocity‐shear combinations, which are unique to the scaffold used in the experiment and do not provide additional information on the individual effects of shear stress and velocity. A method to circumvent this challenge will be discussed in Section 5—“Future challenges and strategy”.

### 4.2 Semirandom architectures

Most traditional scaffold manufacturing techniques can create only semirandom architectures, that is, structures with wide distributions of pores and pores interconnections shapes and sizes, commonly associated with significant heterogeneities of the macroporous network.

#### 4.2.1 Heterogeneity issues

Depending on the properties of these heterogeneities (e.g., disparity with mean value and prevalence), scaffold “average” declared properties (cf. Section 2.2) may no longer correlate with the actual flow effects. Indeed, similar to electrical currents, fluids tend to flow through the paths of least resistance. This resistance corresponds to the ratio between the pressure gap between the inlet and the outlet (i.e., pressure drop *∆P*) and the flowrate (*Q*). In a pipe of length *L* and diameter *d*, the resistance *R* is given by the following equations:

(7) R=∆P/Q,

(8) $$R=µL/2\pi d^{4}.$$

**Figure 8:**
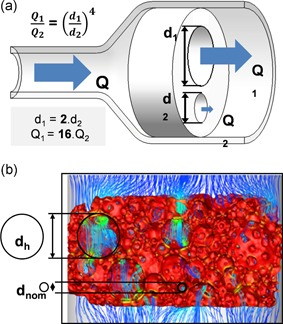
Flowrate distribution vs pore size homogeneity. (a) Illustration of the nonproportional flowrate distribution between two channels of different diameters (Equation (9)). (b) Simulated flow streamlines across a gel cast hydroxyapatite scaffold. Although the nominal pore diameter (*d*_nom_) is 270 µm, a significant portion of the flow is diverted by a few pores (heterogeneities) with a higher diameter (*d*_h_) (from Maes et al., 2012)

For a given pressure drop, the flowrate distribution between two possible pathways of resistance *R*_1_ and *R*_2_ (Figure 8a) is then given by the relationship:

(9) $$\frac{Q_{1}}{Q_{2}}={\frac{R_{2}}{R_{1}}=\left(\frac{d_{1}}{d_{2}}\right)}^{4}.$$

As channel diameter ratios are raised to power 4, even small heterogeneities in pore and pore interconnection sizes can cause significant flow redistributions in a scaffold and redefine the local cell environment.

In Maes et al. (2012), 4‐mm thick scaffolds manufactured by gel casting have a declared pore size of 270 µm. However, whole scaffold µ‐CT revealed the presence of macropores spanning upto 1.6 mm, that is, six times the declared 270 µm pore size and 43% of the scaffold total height. The simulated streamlines clearly show that preferential flow pathways are defined by these macropores, while the rest of the scaffold is comparatively undersupplied (Figure 8b). In those conditions, the mechanical environment is not defined by the specified scaffold properties but by the uncontrolled heterogeneities associated with the manufacturing process.

#### 4.2.2 Distribution issues

The appeal of some irregular structures is in the way they mimic the complexity of the physiological environment. However, random and complex structures are ineffective when trying to understand cell responses to specific stimuli. Most current monitoring techniques (e.g., alkaline phosphatase activity, ARN, and bone‐specific protein expression) allow only the study of cell populations as a whole, thus averaging out the effects of the local flow ranges found across a scaffold (Voronov, VanGordon, Sikavitsas, & Papavassiliou, 2010). Therefore, scaffolds generating broad distributions of shear stress and velocity values make it impossible to identify whether a narrower range of stimuli is responsible for the observed cell response. These distributions depend on the scaffold architectural properties (Boschetti, Raimondi, Migliavacca, & Dubini, 2006); thus, these distributions are unique to each batch of scaffolds or to each scaffold, depending on the reliability of the manufacturing process. In a simulation run by Jungreuthmayer et al. (2009) in a freeze dried collagen‐GAG scaffold, the calculated shear stresses were unevenly scattered across almost two orders of magnitude (0–80 mPa; Jungreuthmayer et al., 2009). In this example, there is no way to know if the observed cell response is due to the cell population submitted to shear stresses in the 5–10 mPa or 40–50 mPa range.

In Liu, Han, Hedrick, Modarres‐Sadeghi, and Lynch (2018), computational fluid dynamics (CFD) are adequately used to confirm that distributions of shear stress and fluid velocity remain constant in various subvolumes of salt‐leached PLG‐HA bone‐mimicking scaffolds. Nevertheless, the calculated shear stress values are distributed from 0.01 to 2,200 mPa, and the fluid velocity ranges from 0 to 4,260 µm/s.

Furthermore, neither mean nor median values, which are often used to describe mechanical environments, permit the discrimination of the range of stimuli responsible for a given osteogenic response. In Jungreuthmayer et al., the mean shear stress value of 19 mPa concerns only a small percentage of the cell population (10.4% in the 16–20 mPa range); therefore, it does not provide additional insights into the optimal ranges of shear stress.

### 4.3 Goldstein approximation

#### 4.3.1 Presentation

**Figure 9:**
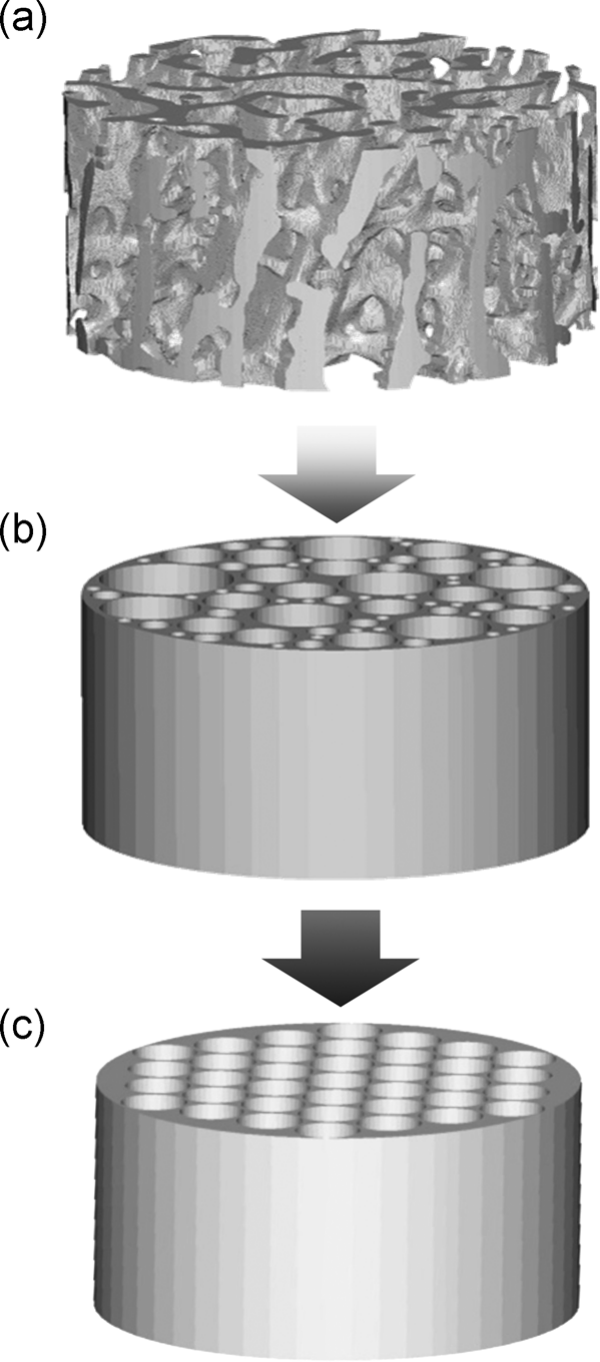
Principle of Goldstein approximation. Illustration of the cylindrical channel approximation from Goldstein et al. (2001). (a) represents a cylindrical section of rat trabecular bone. In (b), the pore networks are simplified to cylindrical parallel channels of various diameters, representing the physiological size distribution of the trabecular bone macroporous network. In (c), those channels are considered to have the same diameter, taking the sample average pore size as reference. The flow‐induced shear stress in a is estimated by applying Equation (6) τ = (8.μ.Q)/(d.A.p) to (c)

To estimate fluid circulation velocity and shear stress levels inside the scaffold from the flowrate value and scaffold properties, Goldstein et al. (2001) introduced a simplified scaffold model. In this approximation, the complex scaffold geometry is simplified by reducing the interconnected pore network to a bundle of parallel, cylindrical channels whose diameters are equal to the scaffold average pore size (Figure 9; Grayson et al., 2008). This model allows the direct use of the simple relationships between the setup parameters and resulting flow effects presented in Section 2.1 (cf. Eq. (1) to (5)).

#### 4.3.2 Usefulness and limitations

In a comprehensive list of 48 perfusion bioreactor‐based studies edited from the most cited reviews in the field (Gardel et al., 2014; McCoy & O'Brien, 2010; Yeatts & Fisher, 2011), two‐thirds of the studies limited their experiments to comparing static culturing with perfused culturing. In addition, most of the 16 studies that did take shear stresses into account relied on the Goldstein model. Thus, this approximation played a central part in shaping the scientific landscape surrounding perfusion bioreactors and bone mechanotransduction studies.

By introducing and popularizing an easily obtainable shear stress estimation, the Rice University team was among the first and most vocal to note the need to determine effective flow effects across different 3D structures and promoted a sounder methodology by encompassing a range of flow and scaffold‐related parameters. However, this approximation offers only an estimation of average flow conditions based on the average theoretical scaffold properties; therefore, it provides limited insight into the actual mechanical environment of the cells (cf. Section 4.2).

**Figure 10:**
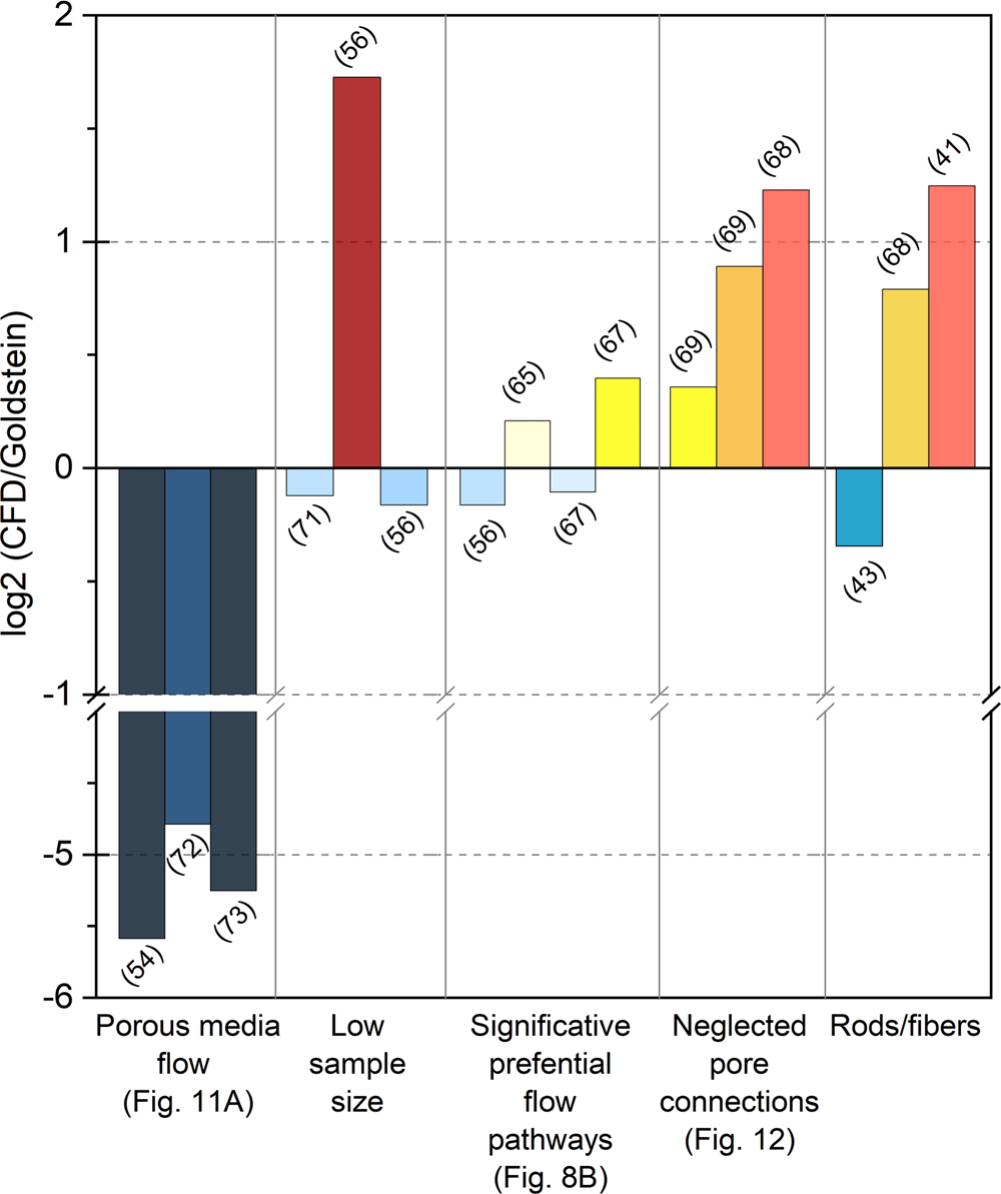
Goldstein approximation robustness. Histogram of the log2 ratio of the computational fluid dynamics (CFD)‐generated shear stress values over the average shear stress in the scaffold predicted by the Goldstein approximation. Log2 values vary between −5.5 and 1.8, indicating that depending on the study, the Goldstein approximation gives shear stress values ranging from 45 times lower (Kleinhans et al., 2015) to three times higher (McCoy et al., 2012) than those predicted by CFD

Currently, shear stress values can be predicted through CFD through 3D models computed from scaffolds (Boschetti et al., 2006; Cioffi, Boschetti, Raimondi, & Dubini, 2006; Jungreuthmayer et al., 2009; Maes et al., 2012; McCoy et al., 2012; VanGordon et al., 2011). The steadily increasing computational power and availability of high‐end CFD software make these simulations the most reliable tool at our disposal to assess mechanical constraints in complex geometries. By comparing shear stresses predicted by CFD data with corresponding mean shear stress values predicted by the Goldstein approximation, we found that although suitable in some studies (Cioffi et al., 2006; Jungreuthmayer et al., 2009; Maes et al., 2012), the approximation could also generate heavily inaccurate results, as highlighted in Figure 10. The links between the approximation accuracy and the scaffold internal architecture are developed in Section 4.3.3.

#### 4.3.3 Discrepancies in shear stress predictions and the key role of the macroporous network

**Figure 11:**
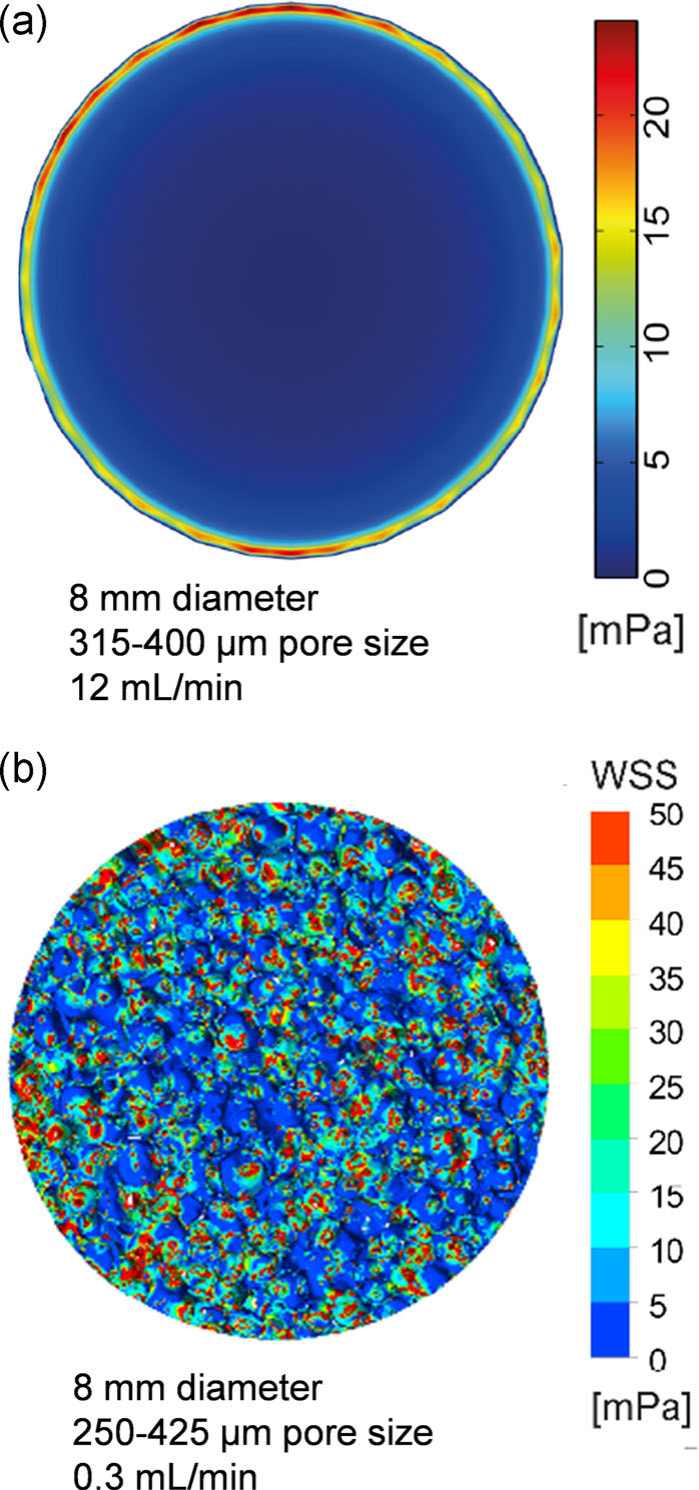
Limit of the homogeneous porous media flow (HPMF) model. (a) Wall shear stress map at the transverse cut plane (50%) in an 8‐mm diameter salt‐leached scaffold (pore size 315–400 µm, porosity 55%) for a 12 ml/min flowrate, calculated with HPMF (from Vetsch et al., 2017). (b) Wall shear stress map at the transverse cut plane (50%) in an 8 mm diameter salt‐leached scaffold (pore size 250–425 µm, porosity 80%) for a 0.3 ml/min flowrate, calculated with finite element analysis based on CFD using the actual scaffold geometry (from Liu et al., 2018)

The homogeneous porous media flow (HPMF) model, which considers the scaffold as a homogeneous permeable solid, has been used in recent studies (Kleinhans et al., 2015; Vetsch, Betts, Muller, & Hofmann, 2017) to encompass scaffold hydraulic properties (e.g., porosity and permeability) while reducing the complexity of the simulation. Figure 11a shows the shear stress distribution at the median transverse cut plane predicted by this model for a flowrate of 12 ml/min (Vetsch et al., 2017). Figure 11b shows the shear stress distribution in a similar scaffold generated by finite element analysis (FEA) based on the actual scaffold geometry for a lower flowrate of 0.3 ml/min. With similar architectural features, shear stress should be proportionally higher for a 12 ml/min flowrate than for 0.3 ml/min (Equation (5)). However, the shear stress values obtained with the porous media flow model are significantly lower and essentially inconsistent with other FEA studies using actual scaffold geometries in similar flow conditions (Cioffi et al., 2006; Jungreuthmayer et al., 2009; Maes et al., 2012; VanGordon et al., 2011). This effect is confirmed in different studies (Egger et al., 2017; Kleinhans et al., 2015) where the use of the HPMF model also led to significantly lower shear stress values than expected (Figure 10). In addition, this model suppresses valuable information by canceling out local shear stress concentrations within the scaffold and overestimates the radial shear stress gradient at the scaffold border, as shown in Figure 11a. For those reasons, we advise against the use of HPMF models in BTE applications.

**Figure 12:**
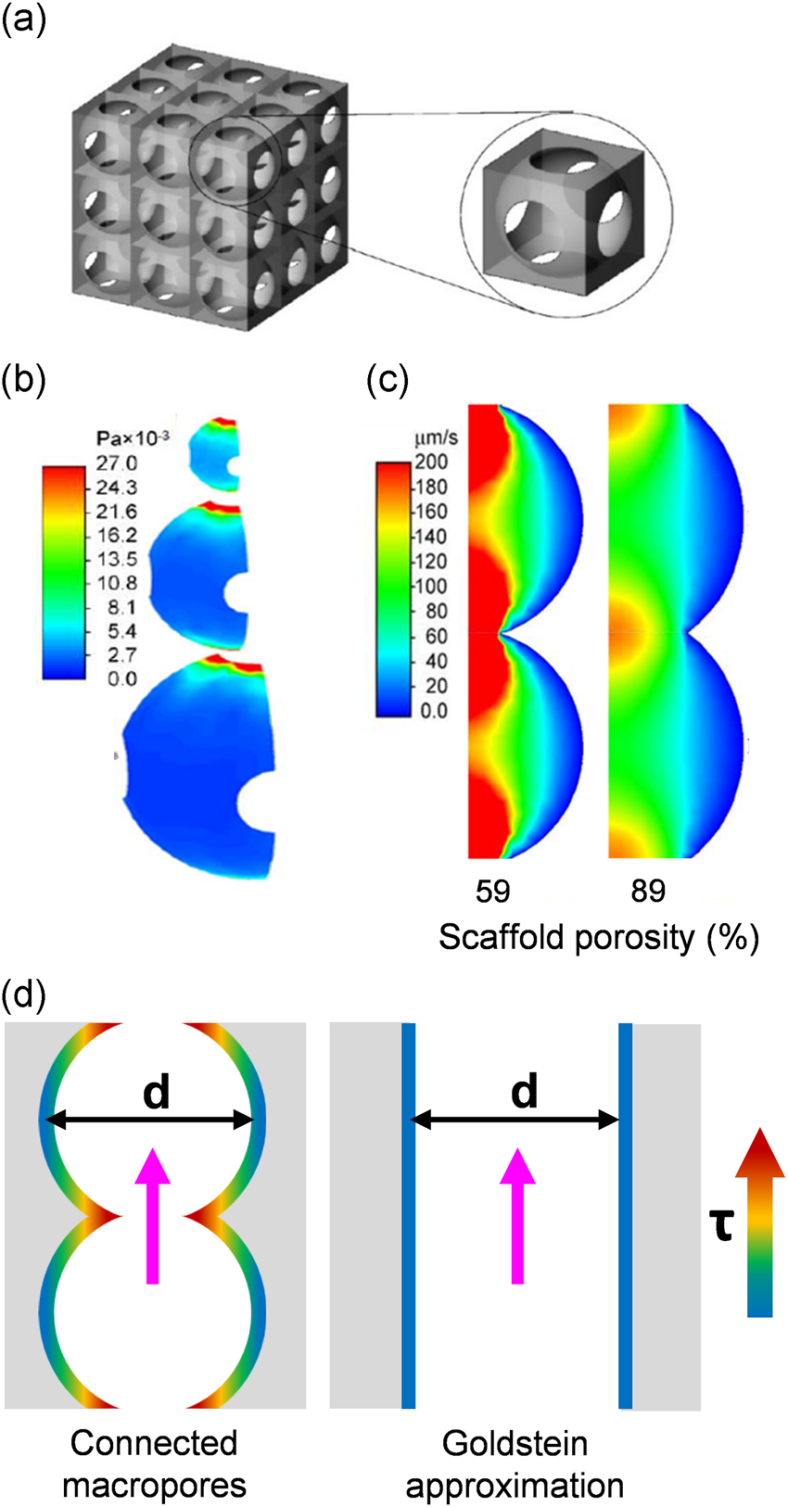
Boschetti pore model. (a) Presentation of the geometrical pore model used in Boschetti et al. (2006). (b) Shear stress maps for different pore sizes (50, 100, and 150 µm) showing the maximal shear stress values at the pore interconnections. (c) Fluid velocity maps show that narrower interconnections compared to the pore diameter generate higher speeds at the interconnections. In Boschetti et al., this effect is erroneously associated with the porosity percentage. (d) Comparison of shear stress repartition in interconnected pores and cylindrical channels of the same diameter *d*, showing why the Goldstein approximation may consistently misestimate shear stress

Boschetti et al. simulated wall shear stresses for multiple combinations of pore sizes and porosity in a pattern‐based 3D model of interconnected spherical macropores, which was inspired by scaffolds fabricated with porogen or salt‐leaching methods. The vertical stacking of constant diameter pores generates parallel cylinder‐like volumes (Figure 12a), allowing a direct comparison with the Goldstein cylindrical model. Configurations in which pore interconnection diameters are close to the pore sizes are geometrically close to a cylinder and logically show a good degree of correlation with the Goldstein approximation (Figure 10). However, tighter interconnections generate stronger shear stress peaks, which correspondingly increase the average shear stress inside the scaffolds (Figure 12b–d). As pore interconnections are not considered in the cylindrical model, using this approximation to calculate shear stress in particle‐leached scaffolds may consistently underestimate the actual average shear stresses in those structures (Boschetti et al., 2006; McCoy et al., 2012; VanGordon et al., 2011).

Studies for which the Goldstein approximation is consistent with the CFD results present distinctive characteristics (Figure 10). In Cioffi et al. (2006), the CFD simulation involves applying the inlet velocity profile to only three isolated pores, which is not representative of the complex flow environment inside heterogeneous scaffolds and neglects the role of pore interconnections. Similarly, in McCoy et al. (2012), the simulation is run on a 360 µm subvolume for an average pore size of 320 µm, limiting its relevance. Moreover, in both Maes et al. (2012) and Jungreuthmayer et al. (2009), presented flow profiles in interconnected macroporous networks show marked preferential pathways diverting flow from the rest of the scaffold and subsequently decreasing the average shear stress to a range consistent with the cylindrical model.

**Figure 13:**
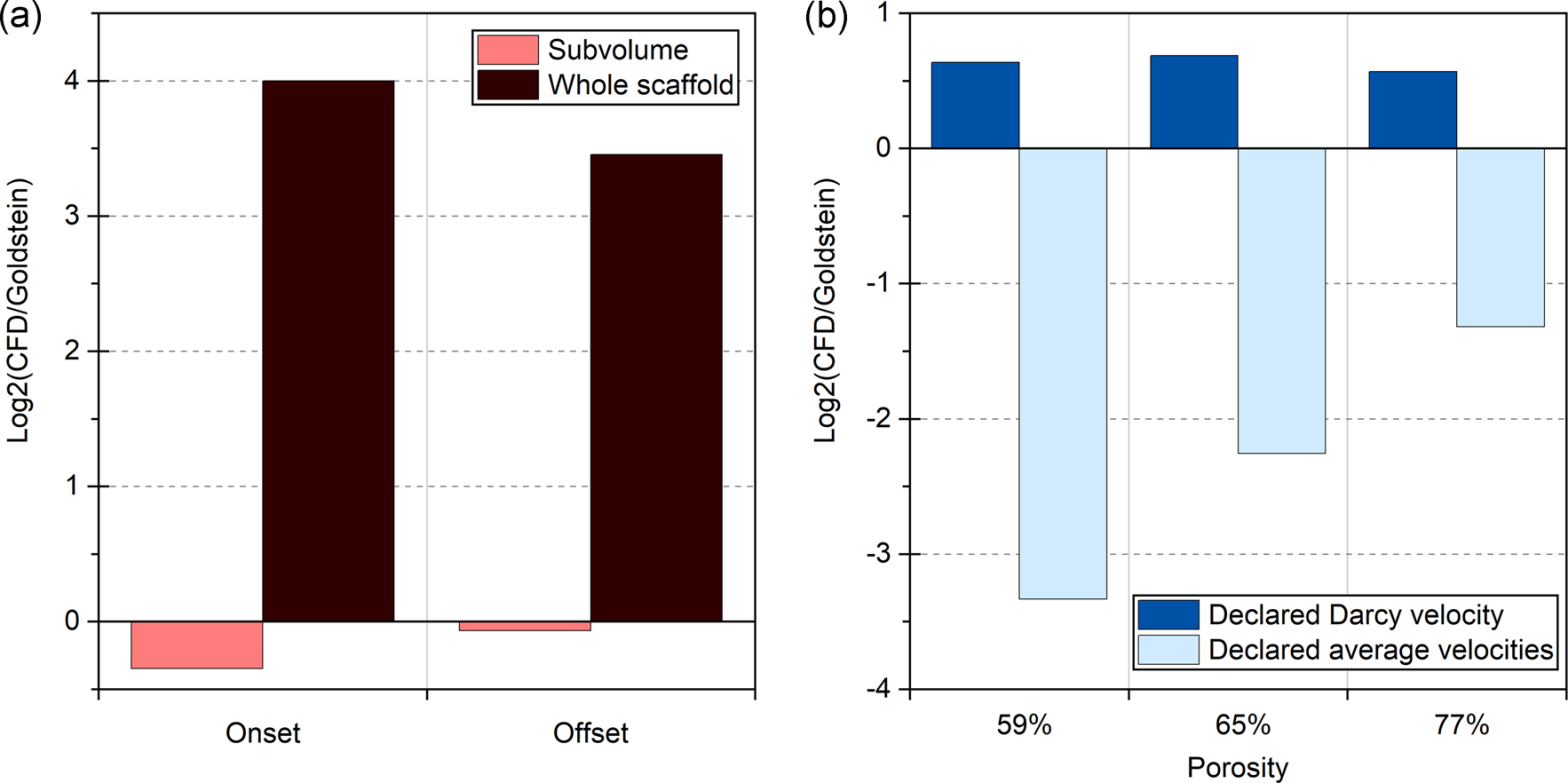
Significance of the reported data. (a) Differences between Goldstein and CFD predictions for shear stress (expressed as log2 [CFD/Goldstein]) in offset and onset robocasted scaffolds for a 1 ml/min flowrate (Allori et al., 2016). In the CFD simulation, this 1 ml/min flowrate is applied to a scaffold subvolume, while it is applied to a whole scaffold in the actual bioreactor. (b) Differences between Goldstein and CFD predictions for shear stress (expressed as log2 [CFD/Goldstein]) in scaffolds with different porosities and macroporous features. Using the declared average velocities instead of the Darcy scale velocity leads to strong discrepancies. CFD, computational fluid dynamics

Furthermore, the parameters used in the Goldstein approximation, such as the “pore diameter”, are sometimes difficult to reconcile with scaffold features (cf. Section 2.2.3). In Allori et al., pores are formed by perpendicular rod stacks. Depending on the geometrical parameter used to define pore size, its declared value in the study varies from 105 to 350 µm. Moreover, in the CFD analysis, a 1 ml/min flowrate is applied to a model subvolume, which is not equivalent to 1 ml/min perfusion of the whole scaffolds, thus diminishing the experimental relevance of the shear stress values obtained (Figure 13a). Boschetti et al. present conflicting values of Darcy scale velocity and average velocity inside the scaffold, strongly impacting the correlation with CFD predicted shear stresses depending on the value used (Figure 13b). In addition, depending on the study, different viscosity values are declared for similar culture media, from 0.78 (Bacabac et al., 2005) to 1.45 (Cruel et al., 2015; Liu et al., 2018; Olivares, Marshal, Planell, & Lacroix, 2009; Zhao, Vaughan, & McNamara, 2016), creating a strong bias in calculated shear stress values for both CFD analysis and the simplified mathematical approach.

As the Goldstein approximation seemed to be the most prevalent source of the seldom declared shear stress values in the literature until a few years ago, the current literature relies on sparse and possibly unreliable data.

### 4.4 Other causes of interference in the interpretation of results

In addition to altered flow effects, sources of variability in cell behavior can stem from other scaffold architectural features as well as the intrinsic chemical properties of the scaffolds and culture media or mechanical stimulation methods. The significant impact of each of these features on in vitro bone growth has been well documented, but it seems to be generally overlooked by teams with unrelated specializations.

#### 4.4.1 Scaffold architectural features and biological response

Beyond altered flow profiles (cf. Section 2), different levels of architecture defined by the manufacturing process significantly affect cell behavior (Li, Wang, Xing, Wang, & Luo, 2016; Marchat & Champion, 2017; Saiz, Zimmermann, Lee, Wegst, & Tomsia, 2013).

Apart from the permeability, mainly controlled by the macroscopic porous network, the size and geometry of macropores and interconnections were also proved to influence cell colonization, tissue growth and osteogenesis, for example, concave surfaces being greater for tissue growth, osteogenesis and microcapillary‐like structure self‐assembly than convex surfaces (Bianchi et al., 2014; Gariboldi, Butler, Best, & Cameron, 2019; Juignet et al., 2017; Rumpler, Woesz, Dunlop, van Dongen, & Fratzl, 2008).

Surface microtopography and micropores are surface structures on a micron scale that are often mistaken for each other. Surface roughness influences the morphology, attachment, proliferation and differentiation of bone cells in vitro (Anselme & Bigerelle, 2005; Sola‐Ruiz, Perez‐Martinez, Martin‐del‐Llano, Carda‐Batalla, & Labaig‐Rueda, 2015; Zhou et al., 2017). In vitro, microporosity results in a larger surface area that is believed to contribute to ion exchange as well as higher bone‐inducing protein adsorption (Gariboldi & Best, 2015; Hannink & Arts, 2011).

#### 4.4.2 Influence of the chemical environment on cell responses to mechanical stimuli

Material surface physicochemical properties (e.g., solubility, wettability, hydrogen potential) and culture medium have a defining impact on in vitro cell behavior and shear stress responses.

##### Shear stress responses conditioned by surface chemistry

Cells approaching an implant material do not make direct contact with its surface but interact with a layer of proteins rapidly adsorbed from the culture medium (Anselme, 2000). Because cells depend on specific proteins for anchorage and extracellular cues, the composition and conformation of adsorbed proteins at the material surface are key mediators of cell behavior (Bouet, Marchat, et al., 2015; Fourel et al., 2016; Streuli, 2009; Wilson, Clegg, Leavesley, & Pearcy, 2005). Material intrinsic properties and particularly surface properties (e.g., surface chemical functional groups) affect the key early event of protein adsorption and subsequent cell adhesion, growth, and differentiation (Anselme, Ponche, & Bigerelle, 2010; Bouet, Marchat, et al., 2015; Vitte, Benoliel, Pierres, & Bongrand, 2004), as well as their response to shear stress (Li, Luo, Huang, et al., 2013, Li, Luo, Xie, et al., 2013; Li et al., 2016). Regarding shear stress responses, Li et al. (2016) demonstrated in 2D systems that the chemical functionalization of glass slides with terminal ‐OH, ‐CH_3_, and ‐NH_2_ groups regulates primary rat osteoblast responses to fluid shear stress.

##### Influence of medium composition

Culture medium ionic environment, pH value, and especially osteogenic supplementation such as dexamethasone, β‐glycerophosphate, and ascorbic acid have been shown to have a significative impact on cell proliferation, differentiation, and overall bone development (Brunner et al., 2010; Monfoulet et al., 2014; Nishimura et al., 2015; Vetsch, Paulsen, Muller, & Hofmann, 2015), but also on cells response to a dynamic perfusion environment. In the presence of osteogenic supplementation human mesenchymal stem cells subjected to shear stress demonstrated significantly stronger increases in growth and alkaline phosphatase activity (Farack et al., 2011). Fetal bovine serum (FBS), another standard supplement of cell culture media, leads to significant differences in experimental outcomes and have in some instances been shown to cause spontaneous mineralization in silk‐fibroin scaffolds, even without cells present (Vetsch, Paulsen, et al., 2015).

Furthermore, FBS‐associated RNA is coisolated with cell culture–derived extracellular RNA and interferes with the downstream RNA analysis. FBS transcripts can also be taken up by cultured cells and affect the results of gene expression profiling technologies (Wei, Batagov, Carter, & Krichevsky, 2016). In secretome profiling, proteins contained in the FBS often mask the proteins secreted by cells, concealing their identification by mass spectrometry (Nonnis et al., 2016). Ill‐defined medium supplementation and recurrent variability in serum batch composition (Brunner et al., 2010) introduce several unknown variables into the cell culture system and might be a major reason why different laboratories are unable to reproduce data published in the literature (Vetsch, Paulsen, et al., 2015).

Additionally, most biological in vitro assays are performed under an atmospheric oxygen concentration (pO_2_  = 20%–21%). However, the native environment of bone stem cells contains much less oxygen (e.g., between 1.3% and 4.2%; Spencer et al., 2014). This “in situ normoxia” (Ivanovic, 2009) was proven to be beneficial for most MSC characteristics, improving growth kinetics, genetic stability, and the expression of molecules by MSCs (Bahsoun, Coopman, Forsyth, & Akam, 2018; Haque, Rahman, Abu Kasim, & Alabsi, 2013; Kwon et al., 2017). As such, the current methodology in bone culture exposes bone cells to nonphysiological and widely hyperoxic environments, resulting in chemical stress and loss of function among cultured cells and highlighting the need to improve the current in vitro culture design (Bahsoun et al., 2018; Hu et al., 2018).

#### 4.4.3 Influence of mechanical stimulus regimen on cell responses

Although most of the above‐mentioned studies used a constant perfusion rate, osteocytes are sensitive to various forms of stimuli in vitro, such as oscillating fluid flow (OFF) stimulation (Batra et al., 2005; Coughlin & Niebur, 2012; Dumas et al., 2009; Kavlock & Goldstein, 2011; Li, Rose, Frances, Sun, & You, 2012; Rauh et al., 2011). Li et al. (2012) undertook a systematic characterization of different OFF parameters on osteocyte activity. The results from this study suggest especially that (a) osteocytes exhibit distinctly different responses to each of the following independent OFF parameters: peak shear stress amplitude, oscillating frequency, and stimulation duration; (b) different mechanotransduction mechanisms likely exist for regulating osteocyte COX‐2 and RANKL/OPG messenger RNA expression; and (c) the effects of each OFF parameter appear to work together in a cumulative manner in regulating osteocyte activity. The introduction of multiple additional parameters (e.g., frequency, amplitude) makes the results provided by these studies more difficult to compare or interpret.

The defining impact of such parameters might raise questions about the widespread use of peristaltic pumps. The characteristics of the pulsatile flow generated by this type of equipment depend on the model and may introduce yet another source of interstudy variability.

Another popular stimulation methodology is to submit cells to the dual effect of perfusion and cyclic mechanical loading of the scaffold; submitting cells to both flow‐induced shear stresses and substrate deformation is an attempt to better reproduce in vivo stimuli (Bouet, Cruel, et al., 2015; David et al., 2008; Dumas et al., 2009; Stops, Heraty, Browne, O'Brien, & McHugh, 2010; Zong ming et al., 2013). Nonetheless, to our best knowledge, fluid movement due to substrate deformation has never been quantified, making the resulting cell responses much more arduous to interpret as the flow patterns remain unknown.

## 5 FUTURE CHALLENGES AND STRATEGY

### 5.1 Defining culture standards

As previously explained, the most common approach since the introduction of 3D perfusion bioreactors has simply been to test combinations of cells and scaffolds and link the observed biological effects to the corresponding flowrate value. Unfortunately, the conclusions of these studies are nongeneralizable as they cannot be related to fluid circulation speed and shear stress values, which are two key factors independently affecting bone cell behaviors and tissue development. Therefore, the first challenging target in the 3D controlled culture system is the determination of the ranges of both shear stress and mass transport values responsible for cell survival and osteogenic stimulation, as outlined in Figure 5c. This challenge can be overcome by the purposeful design of shear stress distribution within the scaffolds at a given velocity. Specifically, narrowing the ranges of both parameters to a homogeneous mechanical environment would allow their direct association with the observed biological response. How a given flowrate will translate into this combination of fluid circulation speed (determining nutrient transport, waste management and paracrine communication mechanisms) and shear stress depends on the bioreactor design and scaffold properties.

#### 5.1.1 System design

In addition to a relevant stimuli regimen, a necessary condition for controlling the scaffold mechanical environment through the flowrate is to ensure that all the medium is actually flowing through the scaffold. The scaffolds could be press‐fitted into custom designed sealing systems to ensure proper perfusion and prevent undesired flow pathways (Du et al., 2015). Leaks can be reduced by adjusting the fluidic circuit to decrease hydrostatic pressure build‐ups and ensuring that components do not deteriorate over the culture duration (Allori et al., 2016). Homogeneous perfusion also requires a homogeneous flow pattern to expose the whole scaffold surface to equivalent flowing conditions. Thus, the bioreactor chamber must be designed in accordance with its expected operating flow rate range to ensure adapted velocity fields throughout the scaffolds. Moreover, depending on the flow conditions, ill‐designed chambers can generate swirls. Swirls can lead to disruptive flow patterns and may lead to medium stagnation, compromising the culture (Freitas, Almeida, & Bartolo, 2014; Vetsch, Hofmann, & Müller, 2015). Therefore, in the development phase, computational simulation of the velocity fields must be performed on the full bioreactor chamber volume to define its suitable design with respect to the expected operating flow parameters and scaffold properties.

#### 5.1.2 Scaffold properties

Scaffold architectural features and the ability to accurately control them play a central role in achieving an exploitable mechanical environment (Choi, Zhang, & Xia, 2010). We find essential to note that randomness often seems to be mistaken for homogeneity (Liu et al., 2018; Maes et al., 2012; Qian, Yuan, Zhimin, & Anchun, 2013). Conventional scaffold manufacturing methods (e.g., particle leaching and fiber meshing) can create only structures with high variability in shape and size within their macroporous network, resulting in “uniformly dispersed” shear stresses and fluid velocities within the scaffolds at best (Liu et al., 2018).

New engineering developments combining computational methods and additive manufacturing (AM) technologies allow for interscaffold repeatability and predictable flow distributions but do not provide a homogeneous mechanical environment unless the scaffolds are designed for this purpose. For instance, in robocasted scaffolds (Figure 4d,f), rods are typically arranged across the flow, exposing different portions of the rods to significantly different shear stress values (Allori et al., 2016; Sonnaert et al., 2017). Moreover, most of the time, flow simulations in AM scaffolds are run with the original 3D model (Allori et al., 2016; Guyot et al., 2014; Sonnaert et al., 2017), neglecting that 3D‐printed scaffolds can display significant intersample variability and deviation from their original design (Marin & Lacroix, 2015).

For generating ranges narrow enough to discriminate the values of shear stress and fluid velocity to which a given cell type is most responsive, an accessible design would comprise cylindrical channels of equivalent diameters arranged alongside the flow direction, actually generating the ideal environment assumed in the Goldstein approximation. The large class of periodic minimal surfaces is also particularly interesting for these in vitro applications. Indeed, triply periodic minimal surfaces ensure a regular macroporous network and can be extensively manipulated, allowing for easier design of shear stress distributions. Moreover, the permeability of these structures is more than tenfold greater than that within a scaffold with random‐pore architecture of comparable porosity and pore size (Melchels et al., 2010).

#### 5.1.3 Disentangling flow effects

As shear stress is proportional to both fluid velocity and viscosity, we can use these parameters to separate the effects of mass transport and shear stress on tissue growth. Increasing the medium viscosity by adding dextran, which does not seem to have an impact on hBMSC cultures at low concentrations (Li, Dai, & Tang, 2008), exposes cultured cells to increased levels of fluid shear stress while maintaining essentially constant chemotransport conditions for nutrient delivery and waste removal. Flowrate and viscosity can also be modified simultaneously to expose cells to various speeds of circulation while maintaining constant shear stress. This strategy established that shear stress and mass transport levels are independent biological stimuli (Li et al., 2009; Sikavitsas, Bancroft, Holtorf, Jansen, & Mikos, 2003). The systematic application of this approach in scaffolds generating narrow and predictable ranges of shear stress and velocity would allow the determination of the optimal fluidic environment for bone growth and more generally, a better understanding of the effects of culture conditions on a given cell type in 3D perfused systems. Since not all cells react in the same way to chemical and mechanical stimuli, it is essential to validate the parameters of the system with the cell type(s) (Bouet, Marchat, et al., 2015) most adapted to the scientific question or intended application. As a key component of the 3D culture system, the cells used have to be thoroughly sorted and selected, for instance with antibodies targeting cell‐specific surface markers (Camilleri et al., 2016; Zhang et al., 2019).

### 5.2 Perspective

The next challenge is the large‐scale implementation of standardized 3D perfused systems, leading to operative platforms for fundamental research or clinical and biomedical applications (Junaid, Mashaghi, Hankemeier, & Vulto, 2017; Pirosa, Gottardi, Alexander, & Tuan, 2018; Tandon, Marolt, Cimetta, & Vunjak‐Novakovic, 2013). These operative platforms will pave the way for a new paradigm for the current procedures of biological testing or the founding principles of preclinical research by achieving significant time, human resource and cost savings over conventional testing as well as the setup of assessment procedures closer to human physiology than animal models.

#### 5.2.1 Live‐monitoring cell behavior

Current monitoring strategies are based on histology, immunohistochemistry, and protein quantification (e.g., enzyme‐linked immunosorbent assay, Western blot analysis, polymerase chain reaction methods). The current state of these technologies provides detailed imaging of various cell mechanisms and high sensitivity to molecules of interest (Kieninger, Weltin, Flamm, & Urban, 2018). However, in addition to being highly time consuming, these techniques are either costly or destructive, greatly limiting the number of data points obtainable in a study.

Nondestructive live‐monitoring techniques (e.g., microscopy, microsensors) would shrink the time associated with traditional monitoring techniques, suppress limitations regarding the number of data points, and greatly reduce the number of scaffolds necessary to obtain statistically significant results over multiple time points. Microsensor systems are already in use regarding culture stable constants, such as O_2_, pH, glucose, and lactate (Kieninger et al., 2018; Zhang et al., 2017). Live‐monitoring of specific cell activity markers would provide unique insights into cellular interactions within a dynamic mechanical and chemical environment (Kieninger et al., 2018), such as osteoblast, osteocyte, osteoclast, or adipocyte markers (e.g., alkaline phosphatase, osteocalcin, sclerostin, Trap5b, adiponectin, and FABP4; Daniele et al., 2018; Han, Ju, & Geng, 2018; Wedrychowicz, Sztefko, & Starzyk, 2018; Zhu et al., 2018). Among other approaches, organic electrochemical transistor (OECT) technology appears to be a strong candidate for this application (Inal et al., 2017; Khodagholy et al., 2013; Rivnay et al., 2013; Rivnay et al., 2018). Further information about OECTs can be found in Rivnay et al. (2018).

#### 5.2.2 Automation

Reasonably, automation has been repeatedly proposed as a solution for standardization and cost reduction (Martin, Smith, & Wendt, 2009; Martin, Wendt, & Heberer, 2004; Nerem, 2014; Salter et al., 2012; Tandon et al., 2013; Yeatts & Fisher, 2011). However, until recently, process automation was associated with high upfront cost and required specific sets of skills to implement. Currently, the increased availability of programmable commercial modular solutions dedicated to fluidics (e.g., Elvesys®, Fluigent®, Cellix®) and accessible microcontrollers (e.g., Arduino®, BeagleBone®, RaspberryPi®) associated with generic fluidics components (e.g., electrovalves, switches, manifolds) paves the way in every laboratory for easier automation of tasks, such as medium sampling and renewal, or even critical steps such as scaffold cell seeding. Many cell seeding protocols do not grant a homogeneous cell distribution, and involve time consuming procedures and technical handling of the seeded scaffold which places unnecessary stress on cells and increases contamination risks. In contrast, automated bioreactor systems can deliver safe and standardized production of engineered tissue constructs, maximizing prospective scale‐up and cost‐effectiveness in the long term (Martin et al., 2009).

#### 5.2.3 Managing culture evolution

In continuous perfusion studies, the flowrate remains constant for the duration of the experiment. However, as bone growth progresses, the construct initial porosity decreases, and pores either decrease in size or become completely obstructed, modifying the mechanical environment to which cells are exposed over time (cf. Section 2). Considering a constant flowrate into a cylindrical channel of initial diameter d_0_ constricted into a channel of diameter d_t_ after a period (*t*) of ECM deposition, the resulting circulation velocity v_t_ and shear stress at the ECM surface τ_t_ in this very simplified model are given by the following equations, obtained by developing Equations (1) and (5) with a constant Q:

(10) $${\text{v}}_{\text{t}}=\frac{{{\text{d}}_{\text{0}}}^{\text{2}}}{{{\text{d}}_{\text{t}}}^{\text{2}}}\;\text{×}\;{\text{v}}_{\text{0}}\;\text{and}\,{\text{τ}}_{\text{t}}=\frac{{{\text{d}}_{\text{0}}}^{\text{3}}}{{{\text{d}}_{\text{t}}}^{\text{3}}}\times {\text{τ}}_{\text{0}}.$$

In experiments involving significant tissue growth in regard to the scaffold's available space, fixing the flowrate systematically initiates an accelerating increase in fluid velocity and shear stress, potentially offering a partial explanation for why many bone cell populations seem to die down after a few days or weeks in vitro. Although working with a constant perfusion flow rate is the most popular methodology, pumps with integrated pressure control can provide a constant pressure drop. In the simplified model described above, the relations between the evolving fluid velocity, shear stress, and the initial conditions in this configuration are then given by the following equations, obtained by developing Equations (5) and (8)with a constant ΔP:

(11) $${\text{v}}_{\text{t}}=\frac{{{\text{d}}_{\text{t}}}^{\text{2}}}{{{\text{d}}_{\text{0}}}^{\text{2}}}\;\text{×}{\text{v}}_{\text{0}}\;\text{and}\,\;{\text{τ}}_{\text{t}}=\frac{{\text{d}}_{t}}{{\text{d}}_{\text{0}}}\text{×}{\text{τ}}_{\text{0}}.$$

This configuration better emulates in vivo conditions where fluid displacements are dictated by pressure differences between more compressed areas and the rest of the bone volume.

Moreover, these models approximate the ECM surface to a moving solid boundary, whereas interstitial flow within the ECM is a key element intervening in the osteocytic differentiation of embedded cells. Compared to the flow‐induced shear stress at the surface of the ECM, interstitial flow within the ECM generates significantly higher levels of shear stress for embedded cells (Guyot, 2015), which are also more likely to be in a bridged configuration (Figure 7b). In addition, to shear stress, bridged cells are also submitted to drag forces (You et al., 2004), which entail greater levels of deformation. Thus, surface shear stress decreasing proportionally to ECM growth (Equation 11) offers an interesting configuration that should be investigated.

Combined with live cell monitoring and automation, numerical bone growth models (Guyot, 2015), integrating the deposition of ECM and its impact on the macroporous network and resulting flow environment, will help to adjust and maintain optimal flow parameters throughout the culture.

#### 5.2.4 From single culture to multiorgan models

Bone homeostasis, especially bone remodeling, is regulated by crosstalk between (a) bone cells, (b) bone cells and cells of other lineages, and (c) bone and other vital organs (Florencio‐Silva, Sasso, Sasso‐Cerri, Simoes, & Cerri, 2015; Zaidi, Yuen, Sun, & Rosen, 2018). The most minimal in vitro models of bone remodeling fundamentally require the coculture of osteoblasts and osteoclasts (Owen & Reilly, 2018). Existing coculture models combining osteoblasts, osteoclasts and sometimes osteocytes, predominantly in conventional 2D (138) or static 3D (e.g., gels; Vazquez et al., 2014), have provided valuable data on osteoblast‐osteoclast interactions and emphasized the role of osteocytes as sensors and orchestrators of the function of both osteoblasts and osteoclasts (Bouet, Cruel, et al., 2015; Florencio‐Silva et al., 2015; Owen & Reilly, 2018; Zhu et al., 2018).

Physiologically relevant, controlled, dynamic 3D coculture models (Beskardes, Hayden, Glettig, Kaplan, & Gumusderelioglu, 2017; Papadimitropoulos et al., 2011; Pirosa et al., 2018) will be able to recapitulate “facets of in vivo organ function”, as described by Edington et al. (2018). Furthermore, the physiological combination of different healthy or pathologic tissue models by means of microfluidic platforms could recreate, at least partly, the systemic cues that mediate interorgan/tissue crosstalk. Uncovering molecular communications between a bone tissue model and other organs or tissues, such as muscle (Brotto & Bonewald, 2015; Karsenty & Mera, 2018; Maurel, Jahn, & Lara‐Castillo, 2017), pancreas (Faienza et al., 2015; Shirakawa, De Jesus, & Kulkarni, 2017), liver (Collier, 2007), kidney (Vervloet et al., 2014), intestine (Keller & Schinke, 2013), stomach (McCabe & Parameswaran, 2017), thyroid and adrenal glands (Rockville, 2004), and lymphoid tissue (Sato et al., 2013), could contribute to a better understanding of the endocrine functions of human bone tissue (Zaidi et al., 2018), bone remodeling, and associated diseases (Maurel et al., 2017; Owen & Reilly, 2018). These combinations of tissue models could also be used as operative platforms for the evaluation of the efficacy, safety, and toxicity of drug candidates (Edington et al., 2018; Ishida, 2018; Kimura, Sakai, & Fujii, 2018; Tetsuka, Ohbuchi, & Tabata, 2017) and medical devices (Guan et al., 2017), potentially boosting research time and cost efficiency while coming into the scope of the 3Rs principles (replace, reduce, and refine). Although the opportunities offered by these models are game‐changing, published proof‐of‐concept studies and prototypes have yet to switch from technology research to actual biological, clinical, and biomedical applications (Junaid et al., 2017; Kimura et al., 2018).

## 6 CONCLUSION

The osteogenic effects of perfusion flow have been discussed for two decades, with the aim of delivering proper guidelines regarding the adequate parameters for bone tissue growth in 3D perfused systems. In this review, we identified multiple factors contributing to this limitation.

A given flowrate breaks down into different shear stress and circulation velocity levels depending on the scaffold features, independently defining the mechanical and chemical cellular environments. Thus, determining generalizable osteogenic culture conditions will require a shift in focus from determining the optimal flowrate in a given setup to defining the optimal combination of shear stress and circulation velocity for a given cell type. A tighter control over the cell environment can be achieved through replacing commonly used approximations by more rigorous culture and scaffold design including flow simulations ahead the system implementation. When possible, implementing automation will ensure higher degrees of repeatability, lighten the culture workload, and provide exciting perspectives when combined with live monitoring of cell activity.

Achieving control over the cell environment and resulting translatability will provide a solid basis for deepening our understanding of the relations between various culture parameters and biological responses in BTE. Eventually, the understanding of these relations will steer the development of new approaches to bone diseases, replacement, and interactions with other organs.

## CONFLICT OF INTERESTS

The authors declare that there are no conflict of interests.
